# Ploidy influences the functional attributes of de novo lager yeast hybrids

**DOI:** 10.1007/s00253-016-7588-3

**Published:** 2016-05-17

**Authors:** Kristoffer Krogerus, Mikko Arvas, Matteo De Chiara, Frederico Magalhães, Laura Mattinen, Merja Oja, Virve Vidgren, Jia-Xing Yue, Gianni Liti, Brian Gibson

**Affiliations:** VTT Technical Research Centre of Finland, Tietotie 2, P.O. Box 1000, FI-02044 Espoo, Finland; Department of Biotechnology and Chemical Technology, Aalto University, School of Chemical Technology, Kemistintie 1, Aalto, P.O. Box 16100, FI-00076 Espoo, Finland; Institute for Research on Cancer and Ageing of Nice (IRCAN), CNRS UMR 7284, INSERM U1081, University of Nice Sophia Antipolis, 06107 Nice, France; ValiRx Finland Oy, Kiviharjuntie 8, FI-90220 Oulu, Finland

**Keywords:** Lager yeast, *S. eubayanus*, Brewing, Hybrid, Rare mating, Heterosis

## Abstract

The genomes of hybrid organisms, such as lager yeast (*Saccharomyces cerevisiae* × *Saccharomyces eubayanus*), contain orthologous genes, the functionality and effect of which may differ depending on their origin and copy number. How the parental subgenomes in lager yeast contribute to important phenotypic traits such as fermentation performance, aroma production, and stress tolerance remains poorly understood. Here, three de novo lager yeast hybrids with different ploidy levels (allodiploid, allotriploid, and allotetraploid) were generated through hybridization techniques without genetic modification. The hybrids were characterized in fermentations of both high gravity wort (15 °P) and very high gravity wort (25 °P), which were monitored for aroma compound and sugar concentrations. The hybrid strains with higher DNA content performed better during fermentation and produced higher concentrations of flavor-active esters in both worts. The hybrid strains also outperformed both the parent strains. Genome sequencing revealed that several genes related to the formation of flavor-active esters (*ATF1*, *ATF2*¸ *EHT1*, *EEB1*, and *BAT1*) were present in higher copy numbers in the higher ploidy hybrid strains. A direct relationship between gene copy number and transcript level was also observed. The measured ester concentrations and transcript levels also suggest that the functionality of the *S. cerevisiae*- and *S. eubayanus*-derived gene products differs. The results contribute to our understanding of the complex molecular mechanisms that determine phenotypes in lager yeast hybrids and are expected to facilitate targeted strain development through interspecific hybridization.

**Electronic supplementary material:**

The online version of this article (doi:10.1007/s00253-016-7588-3) contains supplementary material, which is available to authorized users.

## Introduction

Interspecific hybridization has shown great potential as a strain development tool for the brewing industry, where the natural hybrid yeast *Saccharomyces pastorianus* is utilized to produce the majority of beer worldwide (Gibson and Liti 2015; Hebly et al. 2015; Krogerus et al. 2015a; Mertens et al. 2015). Hybrid species tend to exhibit superior phenotypic qualities compared to one or both parents, i.e., heterosis or hybrid vigor, and this has also been observed in yeast hybrids, which can exhibit improved fermentation rates, greater stress tolerance, and increases in aroma compound production (Bellon et al. 2011; Chen 2013; Gamero et al. 2013; Hebly et al. 2015; Krogerus et al. 2015a; Mertens et al. 2015; Plech et al. 2014; Snoek et al. 2015; Steensels et al. 2014a). Previous studies (Krogerus et al. 2015a; Mertens et al. 2015) reveal that de novo lager yeast hybrids can outperform their parent strains during fermentation and produce beer with similar or higher concentrations of aroma compounds compared to beers produced with the parent strains.

The molecular mechanisms responsible for heterosis are complex and not fully understood. Traditionally, attempts have been made to explain heterosis with the “dominance” and “overdominance” hypotheses, but recent findings using “omics” approaches have suggested more complex mechanisms: allelic interactions, transcriptional regulation, and epigenetic regulation (Chen 2013; Fu et al. 2015; Lippman and Zamir 2007; Shapira et al. 2014). During interspecific hybridization, alloploidization occurs, and allelic genes inherited from different parental species are typically not identical and have, in many cases, quite different functional properties (Chen 2007). Furthermore, hybrid phenotypes may be affected by gene dosage, as the presence of different gene copy numbers can affect regulation and expression (Chen 2007; Yao et al. 2013).

The natural *S. pastorianus* hybrids, resulting from the hybridization of *Saccharomyces cerevisiae* and *Saccharomyces eubayanus*, have been divided into two groups based on their DNA content, Saaz/Group I and Frohberg/Group II, and these differ functionally in a number of respects (Dunn and Sherlock 2008; Gibson et al. 2013a; Liti et al. 2005). The allotriploid Saaz strains, which have retained proportionally more DNA derived from the *S. eubayanus* parent (Walther et al. 2014), tend to possess fermentation characteristics more similar to *S. eubayanus*, while the allotetraploid Frohberg strains, with proportionally more DNA from the *S. cerevisiae* parent (Walther et al. 2014; Nakao et al. 2009), are phenotypically more similar to *S. cerevisiae* ale strains (Gibson et al. 2013a). Recently, genome sequencing of a range of industrial lager yeast strains revealed chromosome copy number variation among Frohberg strains which seemed to directly influence certain phenotypic differences (Van den Broek et al. 2015). Polyploidy and greater gene copy numbers also tend to increase the ability of microbes to resist environmental stresses, which in brewing could comprise, e.g., high osmotic stress and high alcohol concentrations from very high gravity wort (Chen 2007; Gibson et al. 2007; Gibson 2011; Schoenfelder and Fox 2015; Storchova 2014). This was also reflected in a recent study on lager hybrids, where allotriploid hybrids tended to perform better than allodiploid ones (Mertens et al. 2015). Hence, for de novo lager yeast hybrids, a higher ploidy level and thus greater gene copy number could result in increased performance and stress tolerance.

The main yeast-derived flavor compounds in beer are higher alcohols and esters. Esters especially, with their fruity and floral aromas, are considered to contribute a desirable and vital component of beer flavor (Pires et al. 2014). They are mainly formed during fermentation through intracellular enzymatic condensation reactions between alcohols and acyl-CoA, and are divided into two classes: acetate esters and fatty acid ethyl esters. While ester formation is affected by several environmental factors, such as temperature, pH, precursor availability, oxygen concentration, and yeast growth (Hiralal et al. 2014; Pires et al. 2014; Stribny et al. 2015; Yoshioka and Hashimoto 1981), it is also dependent on the expression and enzyme activities of various transferase-encoding genes: *ATF1* and *ATF2* for acetate esters (Verstrepen et al. 2003; Zhang et al. 2013), and *EHT1* and *EEB1* for fatty acid ethyl esters (Saerens et al. 2006, 2008). The expression levels of *ATF1* and *ATF2* especially seem to be directly correlated with the concentrations of acetate esters in beer (Saerens et al. 2008). In lager yeast, these genes typically occur in two allelic forms, with one derived from the *S. cerevisiae* parent and the other from the *S. eubayanus* parent. Recent gene expression studies on lager yeast have revealed variation in expression and product activity of orthologous genes (Bolat et al. 2013; Gibson et al. 2015; He et al. 2014; Horinouchi et al. 2010), suggesting that aroma formation by de novo lager yeast hybrids may be directly affected by the expression of aroma-related orthologous genes inherited from each parent strain. Also, it is hypothesized that aroma formation is affected by the ploidy level of these hybrids, as increased gene copy numbers typically result in increased expression (Yamada et al. 2010).

Here, we generated lager yeast hybrids with different ploidy levels (allodiploid, allotriploid, and allotetraploid) by crossing an *S. cerevisiae* ale strain with the *S. eubayanus* type strain through either spore-to-spore mating or rare mating (Pérez-Través et al. 2012; Steensels et al. 2014b). The contributions of the respective parental genomes to the hybrid genomes were determined by sequencing. The performance of these hybrids with respect to each other and the parent strains was characterized in 2-L fermentations using 15 and 25 °P wort. The fermenting wort and resulting beers were analyzed for aroma compounds, vicinal diketones, and sugar content, while transcript analysis, viability tests, and flocculation assays were performed on the strains. The aim was to investigate to what extent the DNA content of de novo lager yeast hybrids affects fermentation performance, aroma production, and resistance towards intensification of fermentation conditions. Furthermore, the relationship between gene expression and aroma formation in the strains was elucidated. It is expected that results will facilitate the creation of future hybrid brewing yeasts with specific properties.

## Materials and methods

### Yeast strains

The two parental strains were *S. cerevisiae* VTT-A81062 (VTT Culture Collection, Finland), an industrial ale yeast strain, and the *S. eubayanus* type strain VTT-C12902 (VTT Culture Collection, Finland; deposited as CBS12357 at CBS-KNAW Fungal Biodiversity Centre, Netherlands). The three hybrid strains (A81062 × C12902), i.e., allodiploid, allotriploid, and allotetraploid strains, that were chosen for further characterization were named ‘Hybrid A2’, ‘Hybrid B3’, and ‘Hybrid C4’, respectively. Prior to hybridization, natural auxotrophic mutants (*lys-* and *ura-*) of the parental strains were selected on α-aminoadipic and 5-fluoroorotic acid agar plates, respectively (Boeke et al. 1987; Zaret and Sherman 1985). Auxotrophy was confirmed by the inability to grow on minimal selection agar medium (0.67 % Yeast Nitrogen Base without amino acids, 3 % glycerol, 3 % ethanol, and 2 % agar).

### Generation of interspecific hybrids

The allodiploid interspecific hybrid ‘Hybrid A2’, between a *lys-* isolate of *S. cerevisiae* A81062 and a *ura-* isolate of *S. eubayanus* C12902, was produced using spore-to-spore mating. First, ascospores of the auxotrophic mutants were generated on sporulation agar (1 % potassium acetate, 10 mg L^−1^ lysine and uracil, 2 % agar) as described by Krogerus et al. (2015a). Ascus walls were digested with 1 mg mL^−1^ Zymolyase 100T (US Biological, USA), after which spores from the different parental strains were dissected and placed next to each other on YPD agar plates (1 % yeast extract, 2 % peptone, 2 % glucose, and 2 % agar) using a micromanipulator (Singer Instruments, UK). The plates were incubated at 25 °C for 3 days, after which any emerging colonies were replated on minimal selection agar, and incubated at 25 °C for 5 days. Any colonies emerging on the minimal selection agar were regarded as potential hybrids.

The allotriploid interspecific hybrid ‘Hybrid B3’, between a *ura-* isolate of *S. cerevisiae* A81062 and a *lys-* isolate of *S. eubayanus* C12902, was produced using rare mating. A culture of *S. cerevisiae* A81062 *ura-* was grown overnight at 25 °C by inoculating a single colony into 50 mL of YPM (1 % yeast extract, 2 % peptone, 2 % maltose). The culture was centrifuged at 5000×*g* for 5 min, after which the cells were first washed once and then resuspended in sterile H_2_O to a concentration of 10 g centrifuged wet yeast mass L^−1^. Ascospores of *S. eubayanus* C12902 *lys-* were scraped off the agar into 1 mL sterile reverse-osmosis purified H_2_O in 2 mL Eppendorf tubes. Tubes were centrifuged at 5000×*g* for 5 min and the supernatant was removed. Ascus walls were digested by the addition of 50 μL 1 mg mL^−1^ Zymolyase 100T and incubation at 30 °C for 20 min. Two hundred microliters of sterile H_2_O was then added, and the cells and spores were resuspended by vortexing. One hundred microliters of the resulting suspensions from both parental strains, with complementary auxotrophic markers, was transferred together to 1 mL YPM medium in a sterile 2 mL Eppendorf tube. Tubes were vortexed and incubated statically at 25 °C for 5 days. After incubation, the tubes were centrifuged at 5000×*g* for 5 min and the supernatant was removed. Five hundred microliters of starvation medium (0.1 % yeast extract and 0.1 % glucose) was added, and tubes were incubated for at least 2 h at room temperature. Tubes were then vortexed and 100 μL aliquots were spread onto minimal selection agar (without uracil or lysine). Plates were incubated at 25 °C for 5 days and any colonies emerging on the minimal selection agar were regarded as potential hybrids.

The allotetraploid interspecific hybrid ‘Hybrid C4’, between a *ura-* isolate of *S. cerevisiae* A81062 and a *lys-* isolate of *S. eubayanus* C12902, was also produced using rare mating. Hybrid generation was carried out essentially as described above for the allotriploid hybrid. However, instead of using a suspension of *S. eubayanus* C12902 *lys-* spores, a suspension of vegetative cells was used. A culture of *S. eubayanus* C12902 *lys-* was grown overnight at 25 °C by inoculating a single colony into 50 mL of YPM. The culture was centrifuged at 5000×*g* for 5 min, after which the cells were first washed once and then resuspended in sterile H_2_O to a concentration of 10 g centrifuged wet yeast mass L^−1^.

### Confirmation of hybrid status by PCR and RFLP

The hybrid status of isolates was confirmed by PCR as described in Krogerus et al. (2015a). Briefly, the rDNA ITS region was amplified using the primers ITS1 (5′-TCCGTAGGTGAACCTGCGG-3′) and ITS4 (5′-TCCTCCGCTTATTGATATGC-3′), and amplicons were digested using the HaeIII restriction enzyme (New England BioLabs, USA) as described previously (Pham et al. 2011). Amplification of the *S. eubayanus*-specific *FSY1* gene (amplicon size 228 bp) and the *S. cerevisiae*-specific *MEX67* gene (amplicon size 150 bp) was also performed on the DNA extracted from the hybrid strains using the primers SeubF3 (5′-GTCCCTGTACCAATTTAATATTGCGC-3′), SeubR2 (5′-TTTCACATCTCTTAGTCTTTTCCAGACG-3′), ScerF2 (5′-GCGCTTTACATTCAGATCCCGAG-3′), and ScerR2 (5′-TAAGTTGGTTGTCAGCAAGATTG-3′) as described by Muir et al. (2011) and Pengelly and Wheals (2013).

### DNA content by flow cytometry

Flow cytometry was performed on the yeast strains essentially as described by Haase and Reed (2002). Cells were grown overnight in YPD medium (1 % yeast extract, 2 % peptone, 2 % glucose), and approximately 1 × 10^7^ cells were washed with 1 mL of 50 mM citrate buffer. Cells were then fixed with cold 70 % ethanol and incubated at room temperature for 1 h. Cells were then washed with 50 mM citrate buffer (pH 7.2), resuspended in 50 mM citrate buffer containing 0.25 mg mL^−1^ RNAse A, and incubated overnight at 37 °C. Proteinase K (1 mg mL^−1^) was then added, and cells were incubated for 1 h at 50 °C. Cells were then stained with SYTOX Green (2 μM; Life Technologies, USA), and their DNA content was determined using a FACSAria cytometer (Becton Dickinson, USA). DNA contents were estimated by comparing fluorescence intensities with those of *S. cerevisiae* haploid (CEN.PK113-1A) and diploid (CEN.PK) reference strains. Measurements were performed on duplicate independent yeast cultures, and 100,000 events were collected per sample during flow cytometry.

### Genome sequencing and analysis

In order to create a reference sequence to which hybrid sequences would be compared, *S. cerevisiae* strain A81062 was sequenced by BaseClear (Leiden, Netherlands). In brief, a hybrid approach of PacBio 10 kb genomic library sequencing with a PacBio RSII instrument and Illumina NexteraXT pair-end 150 bp library sequencing with a HiSeq 2500 instrument was carried out. A hybrid assembly of the produced data was also done by BaseClear (Leiden, Netherlands). In brief, Illumina reads were de novo assembled with CLC Genomics Workbench and the assembly aligned to PacBio reads with BLASR (Chaisson and Tesler 2012). Information from this alignment was then used to scaffold the contigs with SSPACE-LongRead scaffolder (Boetzer and Pirovano 2014), and gaps in the assembly were filled with GapFiller (Boetzer and Pirovana 2012).

For subsequent analysis steps, the de novo assembly provided by BaseClear was further reference assembled by Ragout (Kolmogorov et al. 2014) to *S. cerevisiae* S288C genome version R64-2-1 (Engel et al. 2013) in order to combine scaffolds to chromosomes. Finally, the processed *S. cerevisiae* A81062 and *S. eubayanus* FM1318 (a monosporic derivative of C12902; Baker et al. 2015) genomes were concatenated to create a single reference genome. An integrative yeast gene annotation pipeline was set up at Liti Lab (full technical detail will be published separately) in order to combine different existing annotation approaches to form an evidence-leveraged final annotation. RATT (Otto et al. 2011), YGAP (Proux-Wéra et al. 2012), and Maker (Holt and Yandell 2011) were used for gene annotation independently. Then their results were further integrated by EVM (Haas et al. 2008). Proteomes and CDS sequences of several representative sensu stricto species (*S. cerevisiae*, *S. paradoxus*, *S. mikatae*, *S. bayanus*, *S. kudriavzevii*, and *S. eubayanus*) were retrieved according to Scannell et al. (2011) and Baker et al. (2015) and used in our annotation pipeline.

Hybrids A2, B3, and C4 were sequenced by Biomedicum Genomics (Helsinki, Finland). In brief, an Illumina NexteraXT pair-end 150 bp library was prepared for each hybrid and sequencing was carried out with a NextSeq 500 instrument. Pair-end reads from the NextSeq 500 sequencing were quality-analyzed with FastQC (http://www.bioinformatics.babraham.ac.uk/projects/fastqc/) and trimmed and filtered with Skewer (Jiang et al. 2014). Alignment, re-alignment, and variant analysis was carried out using SpeedSeq’s (Chiang et al. 2015) FreeBayes SNP prediction and CNVnator copy number variation prediction modules (Abyzov et al. 2011; Garrison and Marth 2012). SNPs predicted by FreeBayes with less than five left and right aligning reads were discarded. FreeBayes detected two types of SNPs: (a) heterozygous—two different alleles of the SNP are present in equal proportions in the hybrid, or (b) homozygous—a SNP only had an allele different than the reference sequence. Variable copy number regions predicted by CNVnator with higher e-value than 0.001 were discarded. Prior to SpeedSeq variant analysis, alignments were filtered to a minimum MAPQ of 50 with SAMtools (Li et al. 2009). Quality of alignments was assessed with QualiMap (García-Alcalde et al. 2012). In order to exclude repeated regions from the genome during variant analysis, *S. cerevisiae* repetitive regions were retrieved from SGD (Engel et al. 2013) and matched to the concatenated reference genome. Additional copy-number analysis was carried out with cn.MOPS (Klambauer et al. 2012). According to author’s instructions, alignments for cn.MOPS were carried out with Bowtie2, mapping a read to one random best mapping position. Scaffolds shorter than 100,000 bp were discarded. Window size was set to 1000 bp.

In order to count chromosomal copy numbers, the median of read coverage for each nucleotide of a gene was calculated and normalized with sample wise median read coverage of all genes. The median of all genes of a chromosome was then calculated and multiplied by a ploidy specific factor as determined by flow cytometry (allotetraploid Hybrid C4: 2, allotriploid Hybrid B3: 1.5, and allodiploid Hybrid A2: 1) for final chromosome copy numbers. Count analysis was done using R-libraries GenomicRanges, GenomicAlignments, Rsamtools, and GenomicFeatures (Lawrence et al. 2013; Morgan et al. 2010).

### Quantitative PCR for copy number analysis

The relative copy numbers of the *S. cerevisiae-* and *S. eubayanus-*derived *ATF1*, *ATF2*, and *EEB1* genes in the hybrid strains were estimated with quantitative PCR of genomic DNA. DNA was extracted from the strains using a GeneJET Genomic DNA Purification kit (Thermo Scientific, USA) with an additional cell disruption step using acid-washed glass beads (Sigma-Aldrich, Finland). The plasmid pUG66 was used as an internal standard (Gueldener et al. 2002). The species-specific primers (see Table S2 in Supplementary material) were designed using the *S. cerevisiae* A81062 and *S. eubayanus* FM1318 (Baker et al. 2015) reference genomes. Species-specific primers for *EHT1* and *BAT1* could not be obtained and were thus excluded from the analysis. The efficiencies (*E*) of the qPCR assays (ranging from 1.96 to 2.00) for each primer pair were calculated using the formula 10^(−1/*m*)^, where *m* is the slope of the line of the threshold cycle (*C*_*T*_)-versus-log dilution plot of the DNA template (5 pg to 50 ng input DNA) (Pfaffl 2001). The PCRs were performed using a LightCycler® 480 SYBR Green I Master mix (Roche Diagnostics, Switzerland) on a LightCycler® 480 II instrument (Roche Diagnostics, Switzerland) in four independent replicates. The following program was used: pre-incubation (95 °C for 5 min), amplification cycle repeated 45 times (95 °C for 10 s, 60 °C for 10 s, 72 °C for 10 s with a single fluorescence measurement), melting curve program (65–97 °C with continuous fluorescence measurement), and finally a cooling step to 40 °C. Data analysis was performed using the supplied LightCycler® 480 Software, version 1.5 (Roche Diagnostics, Switzerland). The copy numbers of the target genes in the hybrid strains relative to the parent strains were calculated using the formula (Pfaffl 2001):

1 $$ \mathrm{Ratio}=\frac{{\left({E}_{target}\right)}^{\varDelta {C}_{T, target}\left( control- sample\right)}}{{\left({E}_{reference}\right)}^{\varDelta {C}_{T, reference}\left( control- sample\right)}} $$

### Fermentations

The three hybrid and two parental strains were characterized in fermentations performed at 15 °C in both a 15 °P high gravity wort and a 25 °P very high gravity wort. Yeast was propagated essentially as described previously (Krogerus and Gibson 2013a), with the use of a “Generation 0” fermentation prior to the actual experimental fermentations. The experimental fermentations were carried out in duplicate, in 2-L cylindroconical stainless steel fermenting vessels, containing 1.5 L of wort medium. The 15 °P wort (69 g maltose, 24.5 g maltotriose, 21.1 g glucose, and 5.4 g fructose per liter) was produced at the VTT Pilot Brewery from barley malt and had a free amino nitrogen (FAN) content of 372 mg L^−1^. The 25 °P wort (127 g maltose, 45.5 g maltotriose, 33.8 g glucose, and 9.2 g fructose per liter) was produced at the VTT Pilot Brewery from barley malt and Maltax 10 malt extract (Senson Oy, Finland), and had a FAN content of 602 mg L^−1^. Yeast was inoculated at a rate of 15 × 10^6^ and 25 × 10^6^ viable cells mL^−1^ to the 15 and 25 °P worts, respectively. The worts were oxygenated to 15 mg L^−1^ prior to pitching (Oxygen Indicator Model 26073 and Sensor 21158; Orbisphere Laboratories, Switzerland). The fermentations were carried out at 15 °C either until an apparent attenuation of 80 % (corresponding to approximately 6.8 % and 12 % ABV in the 15 and 25 °P fermentations, respectively) was reached, until no change in residual extract was observed for 24 h or for a maximum of 23 days if the preceding criteria were not met.

Wort samples were drawn regularly from the fermentation vessels aseptically and placed directly on ice, after which the yeast was separated from the fermenting wort by centrifugation (9000×*g*, 10 min, 1 °C). Samples for yeast-derived flavor compounds, fermentable sugars, and total diacetyl were drawn as above when 33 % apparent attenuation (approximately 2.8 and 5.2 % ABV in the 15 and 25 °P fermentations, respectively) had been reached, 60 % apparent attenuation (approximately 5.0 and 9.0 % ABV in the 15 and 25 °P fermentations, respectively) had been reached, and from the beer. Yeast viability was measured from the yeast that was collected at the end of the fermentations using a Nucleocounter® YC-100™ (ChemoMetec, Denmark).

Flocculation of the yeast strains was evaluated using a modified Helm’s assay essentially as described by D’Hautcourt and Smart (1999). Cultures recovered from fermentation were washed twice with 0.5 M EDTA (pH 7) to break the cell aggregates and then diluted to an OD_600_ of 0.4. Flocculation was assayed by first washing yeast pellets with 4 mM CaCl_2_·2H_2_O solution and resuspending them in 1 mL of flocculation solution containing 4 mM CaCl_2_·2H_2_O, 6.8 g L^−1 ^ sodium acetate, 4.05 g L^−1^ acetic acid, and 4 % (*v*/*v*) ethanol (pH 4.5). Yeast cells in control tubes were resuspended in 0.5 M EDTA (pH 7). After a sedimentation period of 10 min, samples (200 μL) were taken from just below the meniscus and dispersed in 10 mM EDTA (800 μL). The absorbance at 600 nm was measured, and percentage of flocculation was determined from the difference in absorbance between control and flocculation tubes.

### Ethanol tolerance

The ethanol tolerance during wort fermentations of the parental strains and hybrid strains was tested in small-scale fermentations (35 mL) performed in airlock-capped 50-mL centrifuge tubes. Three worts of 10 °P original extract were prepared by diluting 25 °P all-malt wort with deionized water and ethanol: control (0 % ethanol), 5 % ethanol, and 10 % ethanol. Fermentations were carried out in duplicate at 20 °C and were started by adding 15 × 10^6^ viable cells mL^−1^ to each wort. Here, the higher fermentation temperature was chosen in order to not bias the results. Fermentation progress was monitored by weight loss and final alcohol level (% *v*/*v*). Weight losses during fermentation in the worts supplemented with ethanol were expressed relative to that of the control wort.

### Yeast transcriptional analysis

Samples for yeast transcriptional analysis were taken from the 15 °P fermentations 24 h after pitching, after 33 % apparent attenuation had been reached, and after 60 % apparent attenuation had been reached. The yeast was harvested from the fermentation vessels by anaerobically withdrawing wort containing 50–200 mg fresh mass of yeast. Samples were briefly centrifuged (9000×*g*, 3 min, 1 °C) and yeast pellets were washed with ice-cold RNAse-free (dimethyl pyrocarbonate (DMPC)-treated) water, transferred to tared screw-cap cryovials, and immediately frozen in liquid nitrogen before storage at −80 °C. This sampling procedure took less than 10 min. Transcriptional analysis was performed with the TRAC assay essentially as described earlier (Rautio et al. 2007). Sample tubes were weighed to calculate the fresh yeast mass. Frozen yeast samples were suspended (100–200 mg fresh weight mL^−1^) in lysis buffer (ValiRx Finland Oy, Finland). Yeast was disrupted with 500 μL of acid-washed glass beads (Sigma-Aldrich, Finland) twice in a MagNA Lyzer cell homogenizer (Roche Diagnostics, Switzerland) for 45 s at full speed. Hybridization reaction mixtures (125 μL) contained yeast lysate (100 μg biomass; 100–150 ng polyA RNA), 4 pmol biotinylated oligo(dT) capture probe (Ella Biotech), 0.5 pmol each of labeled detection probe (ValiRx Finland Oy, Finland), 75 μL HybMix (ValiRx Finland Oy, Finland), and 1.5 fmol of ssDNA as internal standard (ValiRx Finland Oy, Finland). The hybridizations were carried out in 96-well PCR plates (ABgene, Epsom, UK) at 60 °C for 60 min. The steps following hybridization, including affinity capture, washing, and elution, were automated with a magnetic bead particle processor KingFisher 96 (Thermo Electron, Vantaa, Finland) in 96-well plates as follows: (1) affinity capture of hybridized RNA targets to 50 μg of streptavidin-coated Sera-Mag SpeedBeads (Thermo Fisher Scientific, USA) for 15 min at room temperature, (2) washing of the beads five times for 1.5 min in 100 μL of Wash Buffer (ValiRx Finland Oy, Finland) at room temperature, and (3) elution of probes with 10 μL of formamide (Applied Biosystems, Foster City, CA, USA) for 10 min at 37 °C. The eluates were analyzed by capillary electrophoresis with an ABI PRISM 3730 Genetic Analyzer (Applied Biosystems, Foster City, CA, USA). To calibrate the separation of the detection probes by size, GeneScan-120LIZ size standard (Applied Biosystems, Foster City, CA, USA) was added to each sample. The identity of the probes was determined by the migration speed and the quantity by the peak area. To minimize non-biological variation in the TRAC assay, the signal intensities measured for the target genes were normalized between samples using the signal measured for the ssDNA internal standard. Oligonucleotide probes (for list, see Table S1 in Supplementary material) were designed and validated as described in previous studies (Gibson et al. 2013b, 2015). The TRAC assay was performed on both orthologues (*S. cerevisiae-* and *S. eubayanus-*derived) of five genes previously reported to contribute to ester formation *ATF1*, *ATF2*, *EHT1*, *EEB1*, and *BAT1* (Lilly et al. 2006; Saerens et al. 2006, 2008; Verstrepen et al. 2003; Zhang et al. 2013).

### Wort and beer analysis

The specific gravity, alcohol level (% *v*/*v*), and pH of samples were determined from the centrifuged and degassed fermentation samples using an Anton Paar Density Metre DMA 5000 M with Alcolyzer Beer ME and pH ME modules (Anton Paar GmbH, Austria).

The yeast dry mass content of the samples (i.e., yeast in suspension) was determined by washing the yeast pellets gained from centrifugation twice with 25 mL deionized H_2_O and then suspending the washed yeast in a total of 6 mL deionized H_2_O. The suspension was then transferred to a pre-weighed porcelain crucible, and was dried overnight at 105 °C and allowed to cool in a desiccator before the change of mass was measured.

Concentrations of fermentable sugars (glucose, fructose, maltose, and maltotriose) were measured by HPLC using a Waters 2695 Separation Module and Waters System Interphase Module liquid chromatograph coupled with a Waters 2414 differential refractometer (Waters Co., Milford, MA, USA). An Aminex HPX-87H Organic Acid Analysis Column (300 × 7.8 mm; Bio-Rad, USA) was equilibrated with 5 mM H_2_SO_4_ (Titrisol, Merck, Germany) in water at 55 °C, and samples were eluted with 5 mM H_2_SO_4_ in water at a 0.3 mL min^−1^ flow rate.

Yeast-derived flavor compounds were determined by headspace gas chromatography with flame ionization detector (HS-GC-FID) analysis. Four-milliliter samples were filtered (0.45 μm) and incubated at 60 °C for 30 min, and then 1 mL of gas phase was injected (split mode; 225 °C; split flow of 30 mL min^−1^) into a gas chromatograph equipped with an FID detector and headspace autosampler (Agilent 7890 Series; Palo Alto, CA, USA). Analytes were separated on a HP-5 capillary column (50 m × 320 μm × 1.05 μm column; Agilent, USA). The carrier gas was helium (constant flow of 1.4 mL min^−1^). The temperature program was 50 °C for 3 min, 10 °C min^−1^ to 100 °C, 5 °C min^−1^ to 140 °C, 15 °C min^−1^ to 260 °C and then isothermal for 1 min. Compounds were identified by comparison with authentic standards and were quantified using standard curves. 1-Butanol was used as internal standard.

Total diacetyl (free and acetohydroxy acid form) in the centrifuged fermentation samples was measured according to Analytica-EBC method 9.10 (European Brewery Convention 2004). Samples were heated to 60 °C and kept at this temperature for 90 min. Heating to 60 °C results in the conversion of α-acetolactate to diacetyl. The samples were then analyzed by headspace gas chromatography using a gas chromatograph equipped with a μECD detector and headspace autosampler (Agilent 7890 Series; Palo Alto, CA, USA). Analytes were separated on a HP-5 capillary column (50 m × 320 μm × 1.05 μm column; Agilent, USA). 2,3-Hexanedione was used as an internal standard.

### Fermentation data analysis and visualization

Statistical analysis was performed with R (http://www.r-project.org/) by using one-way ANOVA and Tukey’s test. Heat maps of the concentrations of yeast-derived flavor compounds in the beers were generated in R based on z-scores. The z-scores (*z*) were calculated as *z* = (*x* − *μ*)/*σ*, where *x* is the concentration of an aroma compound in a particular beer, *μ* is the mean concentration of that aroma compound in all beers, and *σ* is the standard deviation of concentration of that aroma compound in all beers.

Correlations between the maximum transcription level of the monitored genes and the concentrations of aroma compounds in the beers fermented from the 15 °P wort were estimated with multiple linear regression followed by ANOVA to test for significance. The maximum transcription levels were fitted to the concentrations of the aroma compounds as follows:

2 $$ {Y}_i={\beta}_{Sc,ij}\cdot {X}_{Sc,j}+{\beta}_{Se,j}\cdot {X}_{Se,ij}+{\beta}_0 $$

where *Y*_*i*_ is the concentration of aroma compound *i*; *X*_*Sc*,*j*_ and *X*_*Se*,*j*_ are the maximum transcription levels of the *S. cerevisiae*- and *S. eubayanus*-derived orthologues of gene *j*, respectively; and *β*_*Sc*,*ij*_, *β*_*Se*,*ij*_, and *β*_0_ are the regression coefficients.

### Nucleotide sequence accession numbers

The *S. cerevisiae* A81062 reads have been submitted to NCBI-SRA under BioProject number PRJNA301545 and the assembled genome to NCBI-WGS under BioProject number PRJNA301545. The Illumina reads from hybrid strains Hybrid A2, Hybrid B3, and Hybrid C4 have been submitted to NCBI-SRA under BioProject number PRJNA301546.

## Results

### Hybrid generation and confirmation

Interspecific hybrids between the *S. cerevisiae* A81062 and *S. eubayanus* C12902 parent strains were successfully generated using both spore-to-spore mating and rare mating. From spore-to-spore mating, a hybridization frequency of 3.6 % was achieved, while rare mating resulted in the emergence of an average of 381 and 4 colonies (corresponding to hybridization frequencies of approximately 7.6 × 10^−6^ and 1.0 × 10^−7^, respectively) on the selection agar from 1 mL of hybridization culture for allotriploid (C12902 spores and A81062 vegetative cells) and allotetraploid generation (C12902 and A81062 vegetative cells), respectively. Hybrid status of these isolates was confirmed with both ITS-PCR combined with RFLP and amplification of *FSY1* and *MEX67* genes using *S. eubayanus-* and *S. cerevisiae-*specific primers (Fig. S1 in Supplementary material). Three hybrids (Hybrid A2 from spore-to-spore mating, Hybrid B3 from rare-mating with C12902 spores, and Hybrid C4 from rare-mating with vegetative cells) were then selected for further characterization. Flow cytometry confirmed that Hybrid A2 was diploid, Hybrid B3 was triploid, and Hybrid C4 was tetraploid (Fig. S2 in Supplementary material).

The de novo sequencing of the diploid *S. cerevisiae* A81062 resulted into 40 scaffolds which span a total genome size of 12 Mbp. These were further assigned to 16 chromosomes and the mitochondrial genome by reference assembly with Ragout (Kolmogorov et al. 2014). A more detailed description of the *S. cerevisiae* A81062 genome will be published separately. Together with the *S. eubayanus* genome (Baker et al. 2015), these sequences were used as the reference genome for hybrid analysis. Chromosome counts of the hybrid genomes were calculated from the median of normalized median read coverage of each chromosome’s genes (Table 1). Counts of the genes monitored with transcriptional analysis were also estimated based on the median read coverage and found to be equal to the count of their associated chromosome. These gene counts were further supported by qPCR analysis (Table S3 in Supplementary material). Furthermore, this analysis was supported by FreeBayes SNP prediction and CNVnator copy number variation module of the SpeedSeq–pipeline (Figs. S3–S10 in Supplementary material show genome coverage, FreeBayes SNP predictions, and CNVnator copy number variation predictions).

**Table 1:** Chromosome copy numbers of the Hybrid A2, Hybrid B3, and Hybrid C4 strains

Chromosome	Genes located on chromosome		Hybrid A2		Hybrid B3		Hybrid C4	
	*Scer*	*Seub*	*Scer*	*Seub*	*Scer*	*Seub*	*Scer*	*Seub*
I			1	1	2	1	2	2
II	Sc-*EHT1*		**1**	1	**2**	1	**2**	2
III			1	1	1	1	1	2
IV		Se-*EHT1*	1	**1**	2	**1**	2	**2**
V			1	1	2	1	2	2
VI			1	1	2	1	2	2
VII	Sc-*ATF2*	Se*-ATF2*	**1**	**1**	**2**	**1**	**2**	**2**
VIII	Sc-*BAT1*	Se-*ATF1*	**1**	**1**	**2**	**1**	**2**	**2**
IX			1	1	2	1	2	2
X			1	1	2	1	2	1
XI			1	1	2	1	2	2
XII			1	1	2	1	2	2
XIII			1	1	2	1	2	2
XIV			1	1	2	1	2	2
XV	Sc-*ATF1*	Se-*BAT1*	**1**	**1**	**2**	**1**	**2**	**2**
XVI	Sc-*EEB1*	Se-*EEB1*	**1**	**1**	**2**	**1**	**2**	**2**

Bold values depict chromosomes containing genes which have been reported to contribute to ester formation and that here were monitored with transcriptional analysis (Lilly et al. 2006; Saerens et al. 2006, 2008; Verstrepen et al. 2003; Zhang et al. 2013)

*Scer*, *S. cerevisiae*; *Seub*, *S. eubayanus*

The *S. cerevisiae* subgenome chromosomes of the allotetraploid Hybrid C4 and allotriploid Hybrid B3 almost exclusively contain heterozygous SNPs (Figs. S6 and S8 in Supplementary material), which supports the fact that two copies of these chromosomes were present in these hybrids as predicted by read coverage analysis. An exception was chromosome III that contained only homozygous SNPs and that read coverage analysis and CNVnator also detected as a single copy (Table 1). In addition, *S. cerevisiae* subgenome chromosome X appears chimeric based on its SNP distribution in Hybrids C4 and B3. Read coverage analysis revealed that the allodiploid Hybrid A2 contained one set of chromosomes derived from *S. cerevisiae* A81062. This was again supported by the SNPs, which were found to be homozygous within the *S. cerevisiae* subgenome of Hybrid A2 (Table 1 and Fig. S4 in Supplementary material). SNPs in the *S. eubayanus* subgenome are too rare to provide support for chromosome copy numbers. In all three hybrids, only 2 % of the SNPs occur in the *S. eubayanus* subgenome. Accordingly, based on *S. eubayanus* genome data from Bioproject PRJNA264003 (Hebly et al. 2015), we estimate that the *S. cerevisiae* A81062 genome has an almost 200 times higher ratio of heterozygosity than *S. eubayanus* C12902. Read coverage analyses show that the mitochondria are derived from *S. eubayanus* in Hybrid C4 and Hybrid B3 and from *S. cerevisiae* in Hybrid A2 (Fig. S10 in Supplementary material).

### Fermentations in 15 and 25 °P wort

**Fig. 1:**
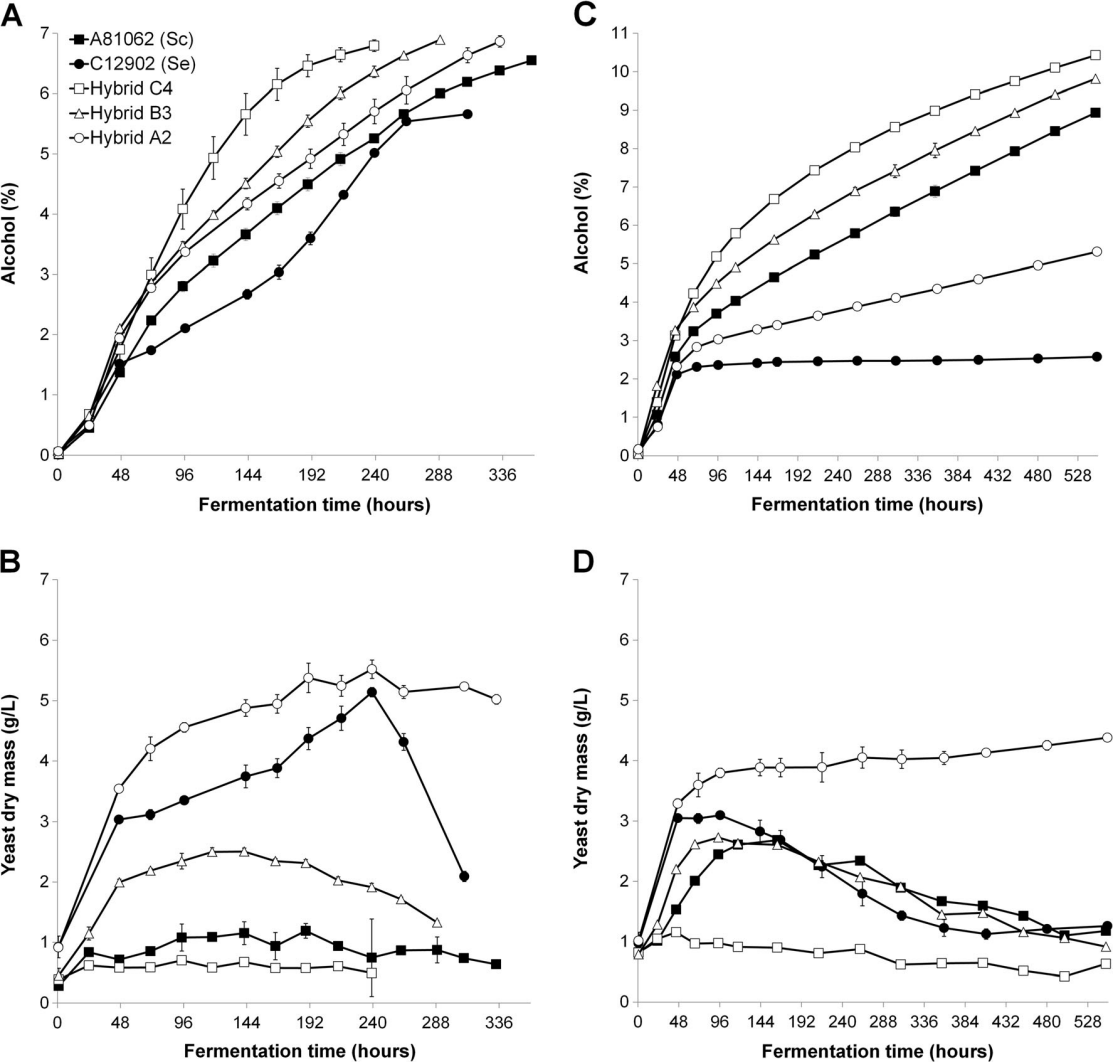
The **a**, **c** alcohol content (% ABV) and **b**, **d** suspended yeast dry mass (g L^−1^) of the 15 and 25 °P worts fermented with the hybrid strains (*white symbols*) and parent strains (*black symbols*), respectively. Values are means from two independent fermentations and *error bars* where visible represent the standard deviation

The wort fermentations revealed that an increased ploidy level in the hybrids was associated with improved fermentation performance. In the 15 °P wort, all three hybrid strains fermented faster than the parent strains throughout the fermentation and had a higher alcohol content when this was measured after 15 days (Fig. 1a and Table 2). In the 25 °P wort, only the allotriploid Hybrid B3 and allotetraploid Hybrid C4 were able to ferment faster than both parent strains (Fig. 1c and Table 2). These also had higher alcohol contents when measured after 23 days of fermentation. While the allodiploid Hybrid A2 fermented more poorly than the *S. cerevisiae* A81062 strain, it did ferment better than *S. eubayanus* C12902 strain. Comparing the hybrid strains, the Hybrid C4 fermented the fastest, followed by Hybrid B3 and finally the Hybrid A2 in both the 15 °P and 25 °P wort. The highest amount of suspended biomass (measured as dry mass) during fermentation was observed for Hybrid A2 in both the 15 and 25 °P wort (Fig. 1b, d). High amounts of suspended biomass were also observed in the *S. eubayanus* C12902 fermentations at 15 °P, but growth was retarded in the harsher conditions of the 25 °P wort. Hybrid B3, Hybrid C4, and the *S. cerevisiae* parent strain showed lower amounts of suspended biomass during fermentation, presumably as a result of their higher flocculation abilities (Table 2). The *S. eubayanus* parent strain showed a rapid drop in suspended biomass at the end of the fermentation in the 15 °P wort despite its low flocculation ability. The beer pH also showed considerable variation (Table 2).

**Table 2:** The parameters of the beers produced from the 15 and 25 °P wort, the flocculation ability and viability of the parent and hybrid strains after fermentation in the 15 and 25 °P wort, as well as the total amount of CO_2_ lost during fermentation of a 10 °P wort supplemented with 5 and 10 % (*v*/*v*) ethanol in relation to the unsupplemented control wort

Yeast strain		A81062	C12902	Hybrid C4	Hybrid B3	Hybrid A2
15 °P	Alcohol (% *v*/*v*)	6.6 (±0.04)^a^	5.7 (±0.01)^b^	6.8 (±0.10)^a^	6.9 (±0.09)^a^	6.9 (±0.10)^a^
	Attenuation (%)	78 (±0.2)^a^	68 (±0.01)^b^	80 (±1.0)^a^	82 (±1.0)^a^	82 (±3.6)^a^
	Maltose (g L^−1^)	15.8 (±0.5)^a^	4.0 (±0.8)^b^	9.0 (±0.6)^c^	4.8 (±0.7)^b^	11.0 (±2.2)^c^
	Maltotriose (g L^−1^)	6.0 (±0.02)^a^	25.9 (±0.6)^b^	7.2 (±0.2)^c^	8.5 (±0.3)^d^	6.6 (±0.1)^a, c^
	pH	4.44 (±0.01)^a^	4.52 (±0.01)^b^	4.68 (±0.01)^c^	4.46 (±0.02)^a^	4.10 (±0.01)^d^
	Yeast viability (%)	89.9 (±1.0)^a^	58.2 (±8.8)^b^	60.7 (±3.0)^b^	87.7 (±0.1)^a^	98.5 (±0.0)^a^
25 °P	Alcohol (% *v*/*v*)	8.9 (±0.22)^a^	2.6 (±0.01)^b^	10.4 (±0.06)^c^	9.8 (±0.25)^c^	5.3 (±0.01)^d^
	Attenuation (%)	58 (±1.6)^a^	17 (±0.2)^b^	67 (±0.6)^c^	64 (±1.7)^c^	32 (±0.3)^d^
	Maltose (g L^−1^)	63.7 (±2.6)^a^	114.2 (±0.8)^b^	39.8 (±1.0)^c^	44.4 (±3.1)^c^	104.4 (±3.7)^d^
	Maltotriose (g L^−1^)	11.0 (±0.9)^a^	44.3 (±1.4)^b^	11.9 (±0.2)^a, c^	13.7 (±1.6)^c^	21.3 (±0.9)^d^
	pH	4.54 (±0.01)^a^	4.71 (±0.01)^b^	4.63 (±0.01)^c^	4.56 (±0.01)^a^	4.49 (±0.01)^d^
	Yeast viability (%)	93.2 (±0.2)^a, b^	0.0 (±0.0)^c^	5.9 (±3.3)^c^	89 (±0.1) ^a^	96.8 (±0.1)^b^
Flocculation ability (%)		94.0 (±0.7)^a^	2.6 (±1.7)^b^	58.6 (±2.1)^c^	73.1 (±5.2)^d^	6.2 (±0.7)^b^
Relative CO_2_ loss in wort with 5 % (*v*/*v*) ethanol (%)		105.5 (±5.2)^a^	58.8 (±7.4)^b^	94.5 (±2.6)^a^	100.4 (±1.3)^a^	59.3 (±7.3)^b^
Relative CO_2_ loss in wort with 10 % (*v*/*v*) ethanol (%)		38.9 (±0.4)^a^	27.3 (±1.3)^b^	27.0 (±3.1)^b^	29.4 (±3.6)^b^	22.9 (±1.4)^b^

Values in the same row with different superscript letters differ significantly (*p* < 0.05). The flocculation abilities are means of three independent assays (standard deviation in parentheses), while beer parameters, viabilities, and relative CO_2_ loss are means from two independent fermentations (standard deviation in parentheses)

*ND* not detected

**Fig. 2:**
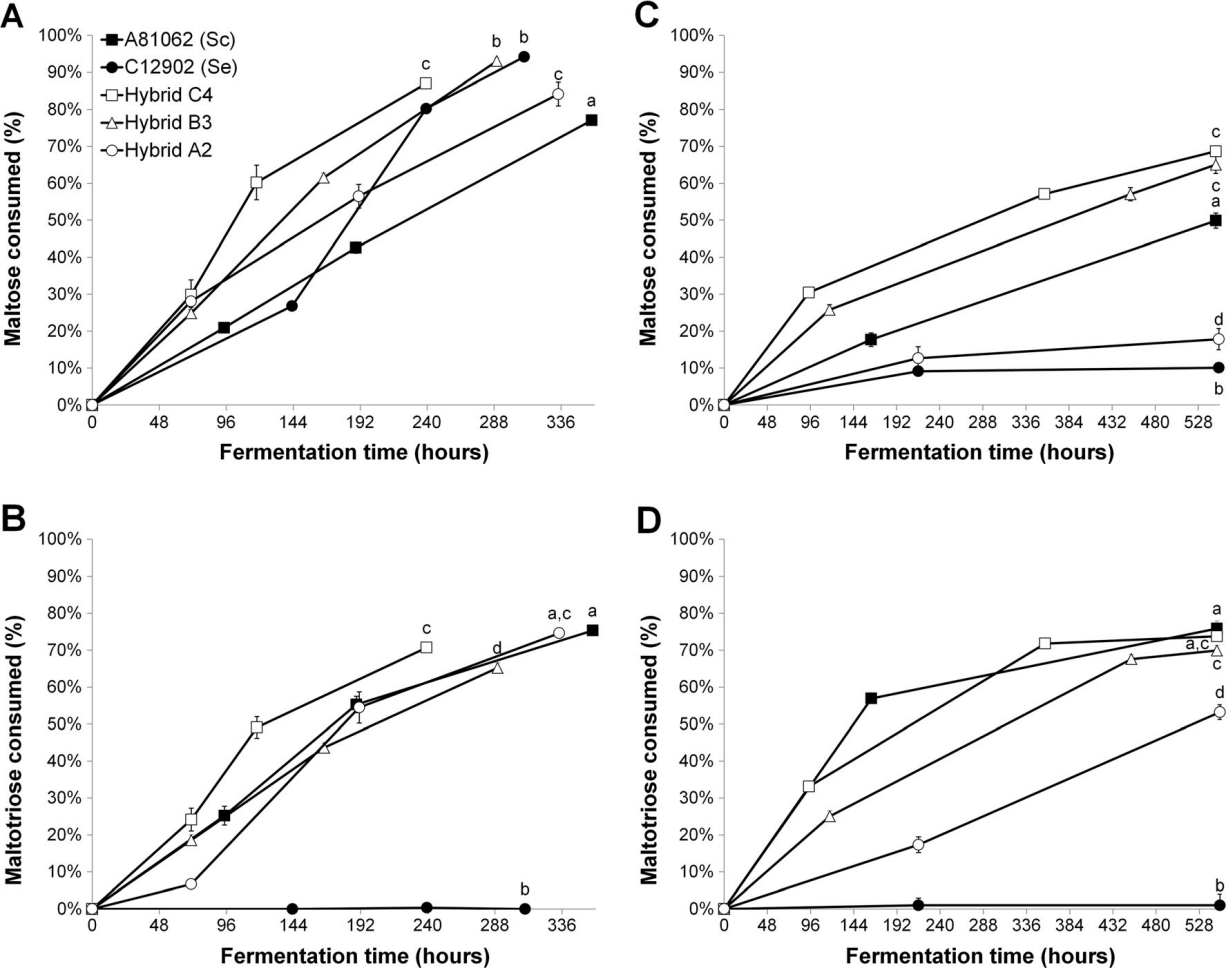
The amount of **a**, **c** maltose (% of concentration in original wort) and **b**, **d** maltotriose (% of concentration in original wort) consumed in the 15 and 25 °P worts fermented with the hybrid strains (*white symbols*) and parent strains (*black symbols*), respectively. Values are means from two independent fermentations and *error bars* where visible represent the standard deviation. Values with different *letters* above the final sampling point differ significantly (*p* < 0.05)

The differences in fermentation rate among the hybrid strains and the parent strains can partly be explained by their sugar consumption during fermentation. In the 15 °P wort, the allotetraploid Hybrid C4 was the strain that used maltose and maltotriose fastest in the first half of fermentation (Fig. 2a, b). The allotriploid Hybrid B3 and allodiploid Hybrid A2 consumed maltotriose at a similar rate throughout fermentation, but the overall fermentation rate of Hybrid B3 was faster as a result of more efficient maltose consumption. The *S. cerevisiae* A81062 parent strain consumed maltotriose at a similar rate to Hybrid B3 and Hybrid A2, but consumed maltose at a lower rate than all three hybrid strains and also the *S. eubayanus* C12902 parent strain in the latter half of fermentation. As was expected based on previous research (Gibson et al. 2013a; Krogerus et al. 2015a), the *S. eubayanus* parent strain was unable to consume maltotriose. No glucose or fructose was detected in any of the samples that were drawn during fermentation, suggesting these sugars were rapidly depleted from the wort. In the 25 °P wort, similar trends were observed, with the strains fermenting the fastest also consuming maltose and maltotriose the fastest (Fig. 2c, d). Hybrid A2 and the *S. eubayanus* strain consumed maltose poorly in the 25 °P wort, resulting in an overall low fermentation rate.

The viability measurements of the yeast harvested from the 15 and 25 °P fermentations revealed that the *S. cerevisiae* A81062 parent strain, allotriploid Hybrid B3, and allodiploid Hybrid A2 were least affected by the intensified fermentation conditions in the 25 °P wort (Table 2). Relatively low viabilities were measured for the allotetraploid Hybrid C4, probably because it had been exposed to higher concentrations of ethanol for a longer time as a result of its faster fermentation. The fermentation assay in worts supplemented with 5 and 10 % ethanol revealed that the *S. cerevisiae* parent strain performed best in the presence of ethanol (Table 2). In the wort supplemented with 5 % ethanol, Hybrid B3 and Hybrid C4 also nearly reached the same fermentation degree (100 %) as in the control wort, while the C12902 parent strain and Hybrid A2 only reached approximately 60 %. In the wort supplemented with 10 % ethanol, all strains performed poorly. However, the *S. cerevisiae* parent strain reached a slightly higher fermentation degree than the other strains (39 vs. 29 %).

### Aroma compounds in the beers

**Fig. 3:**
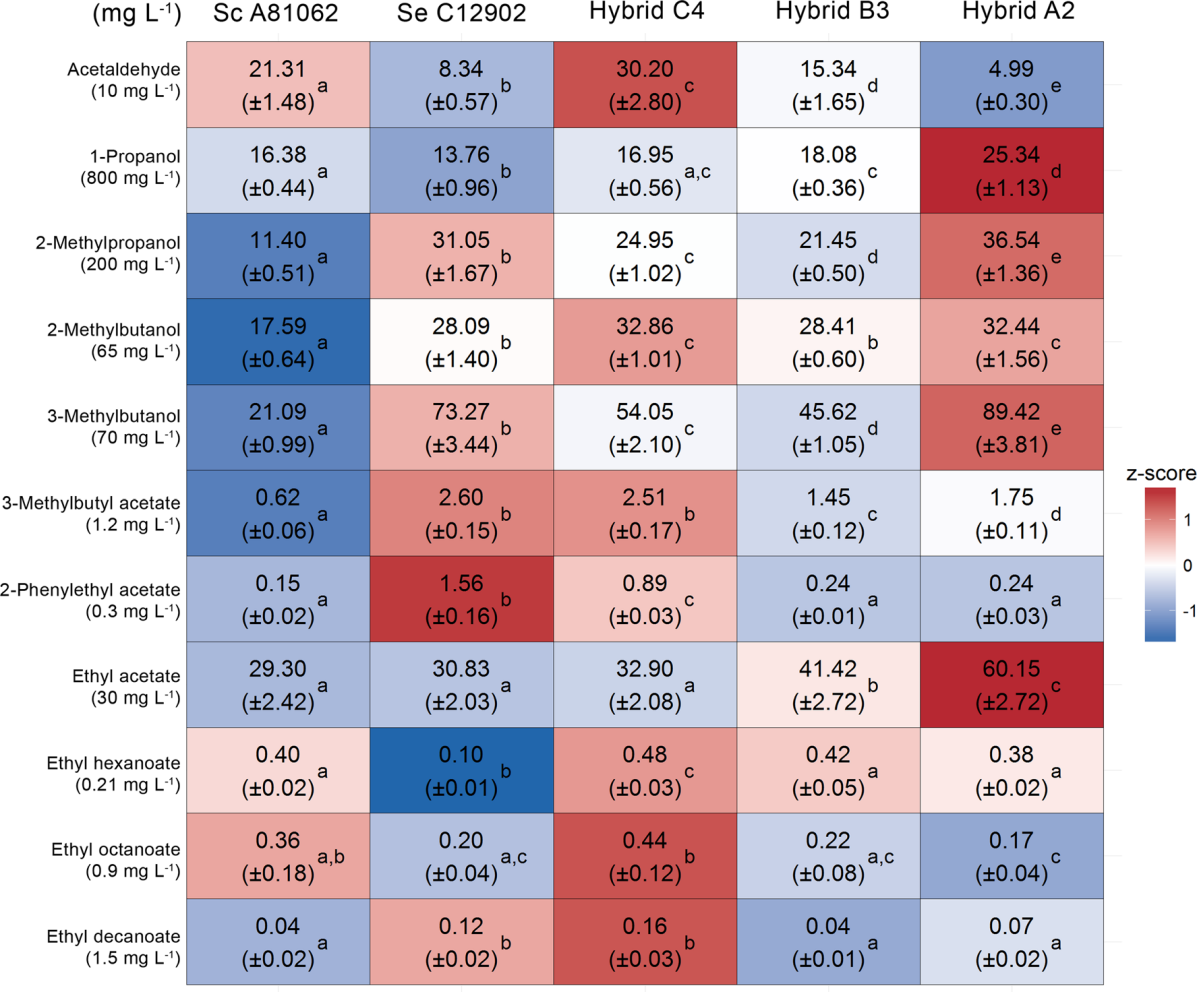
The concentrations (mg L^−1^) of aroma compounds (rows) in the beers fermented from the 15 °P wort with the hybrid and parent strains (columns). The heat map was generated based on the z-scores (*blue* and *red* indicate low and high values, respectively). The values in *parentheses* under the compound names represent the flavor threshold (Meilgaard 1982). Values are means from two independent fermentations (standard deviation in parentheses) and they have not been normalized to the ethanol concentration. Values in the same row with different superscript *letters* (*a–e*) differ significantly (*p* < 0.05)

**Fig. 4:**
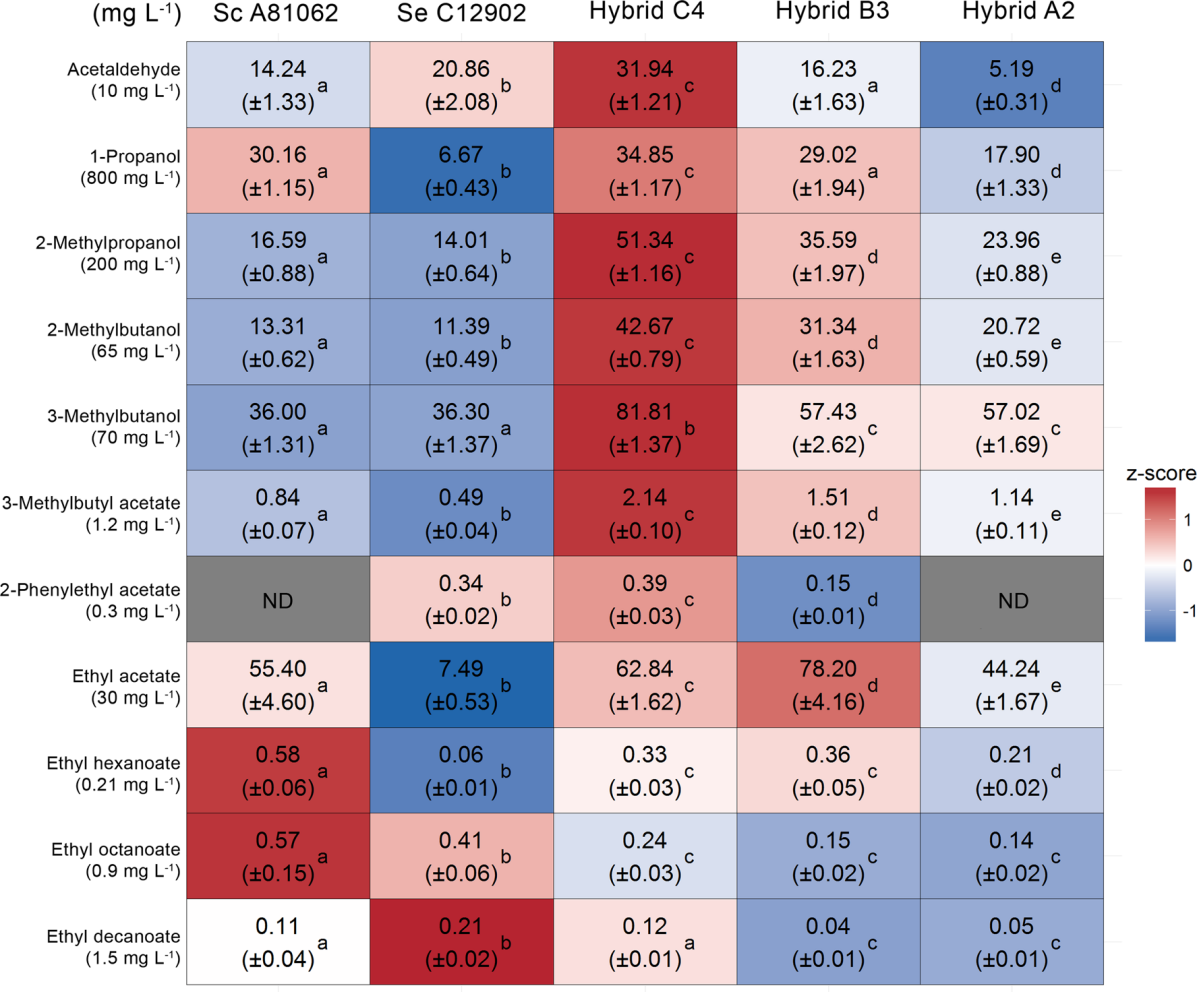
The concentrations (mg L^−1^) of aroma compounds (rows) in the beers fermented from the 25 °P wort with the hybrid and parent strains (columns). The heat map was generated based on the z-scores (*blue* and *red* indicate low and high values, respectively). The values in *parentheses* under the compound names represent the flavor threshold (Meilgaard 1982). Values are means from two independent fermentations (standard deviation in *parentheses*) and they have not been normalized to the ethanol concentration. Values in the same row with different superscript *letters* (*a–e*) differ significantly (*p* < 0.05)

The concentrations of yeast-derived aroma compounds in the beers showed notable variation (Figs. 3 and 4). For higher alcohols and esters, the trends between the different yeast strains remained similar throughout fermentation (Figs. S11 and S12 in the Supplementary material). Of the beers fermented from the 15 °P wort, the allotetraploid Hybrid C4 produced the highest overall concentrations of flavor-active esters, showing a higher concentration of ethyl hexanoate than either parent and as high concentrations of 3-methylbutyl acetate, ethyl octanoate, and ethyl decanoate as the better parent. Comparing the parent strains, *S. cerevisiae* A81062 tended to produce higher concentrations of ethyl esters, while *S. eubayanus* C12902 tended to produce higher concentrations of acetate esters. This was reflected in the aroma profiles of the beers fermented with the hybrid strains and the contribution of the parent genomes in these hybrids. The beers fermented with the allotriploid Hybrid B3, containing proportionally more of the *S. cerevisiae* than the *S. eubayanus* parent genome, contained lower amounts of acetate esters (3-methylbutyl acetate and ethyl acetate) than the beers fermented with the other two hybrids.

In the 25 °P fermentations, the aroma compounds were at similar levels as in the 15 °P fermentations (Fig. 4). As in the 15 °P fermentations, when comparing the hybrid strains, Hybrid C4 produced the beer with the highest concentrations of flavor-active esters (higher concentrations of the acetate esters than either parent strain). Compared to Hybrid C4, Hybrid B3 produced similar concentrations of all ethyl esters, but significantly lower concentrations of 3-methylbutyl acetate and 2-phenylethyl acetate. As a result of the poor fermentations observed with the *S. eubayanus* parent and Hybrid A2, low amounts of flavor-active esters were also observed in the beers produced with these strains. The *S. cerevisiae* parent strain again produced high concentrations of ethyl esters.

**Fig. 5:**
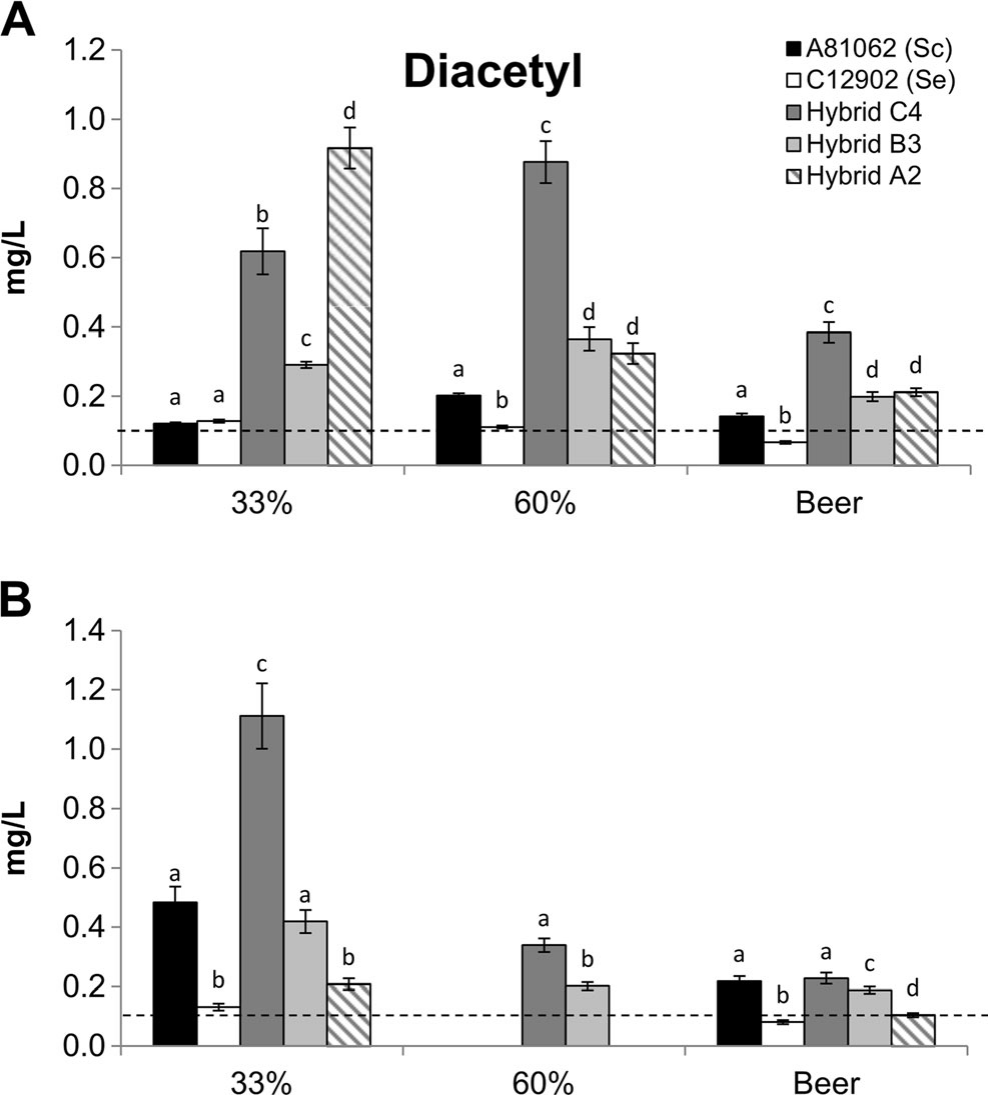
The concentrations of diacetyl in the wort (33 and 60 % attenuation) and beers fermented from the **a** 15 °P and **b** 25 °P wort with the hybrid and parent strains (mg L^−1^). Where visible, the *dashed line* represents the typical flavor threshold (Meilgaard 1982). Values are means from two independent fermentations and *error bars* where visible represent the standard deviation. Values from the same sampling point with different *letters* (*a–d*) above the bars differ significantly (*p* < 0.05)

The concentrations of diacetyl in the wort and beer also showed considerable variation among the parent and hybrid strains in both fermentations (Fig. 5). During the 15 °P fermentation, the parent strains showed low levels of total diacetyl, with a maximum peak of around 200 μg L^−1^ for *S. cerevisiae* A81062. The highest diacetyl peaks, at around 900 μg L^−1^, were observed for Hybrid C4 and Hybrid A2. However, the diacetyl concentration in the beer fermented with Hybrid A2 was only 210 μg L^−1^, while it was 385 μg L^−1^ for Hybrid C4. The diacetyl concentrations of Hybrid B3 were in between those of Hybrid C4 and the parents. During the 25 °P fermentations, the highest diacetyl peak was again observed for Hybrid C4, while the lowest diacetyl levels were again observed for *S. eubayanus*. In contrast to the 15 °P fermentation, relatively low concentrations of diacetyl were observed for Hybrid A2. These are most likely a result of the poor fermentation performance that was observed for this strain in the 25 °P wort.

### Transcriptional analysis

Transcriptional analysis was performed on both orthologues (*S. cerevisiae-* and *S. eubayanus-*derived) of five genes previously reported to contribute to ester formation (Lilly et al. 2006; Saerens et al. 2006, 2008; Verstrepen et al. 2003; Zhang et al. 2013): *ATF1*, *ATF2*, *EHT1*, *EEB1*, and *BAT1* (Table 1). Yeast samples were taken from the 15 °P fermentations at different time points. Positive correlations were observed both between the overall transcription levels and the deduced gene copy numbers, as well as the transcription levels of specific genes and corresponding esters.

**Fig. 6:**
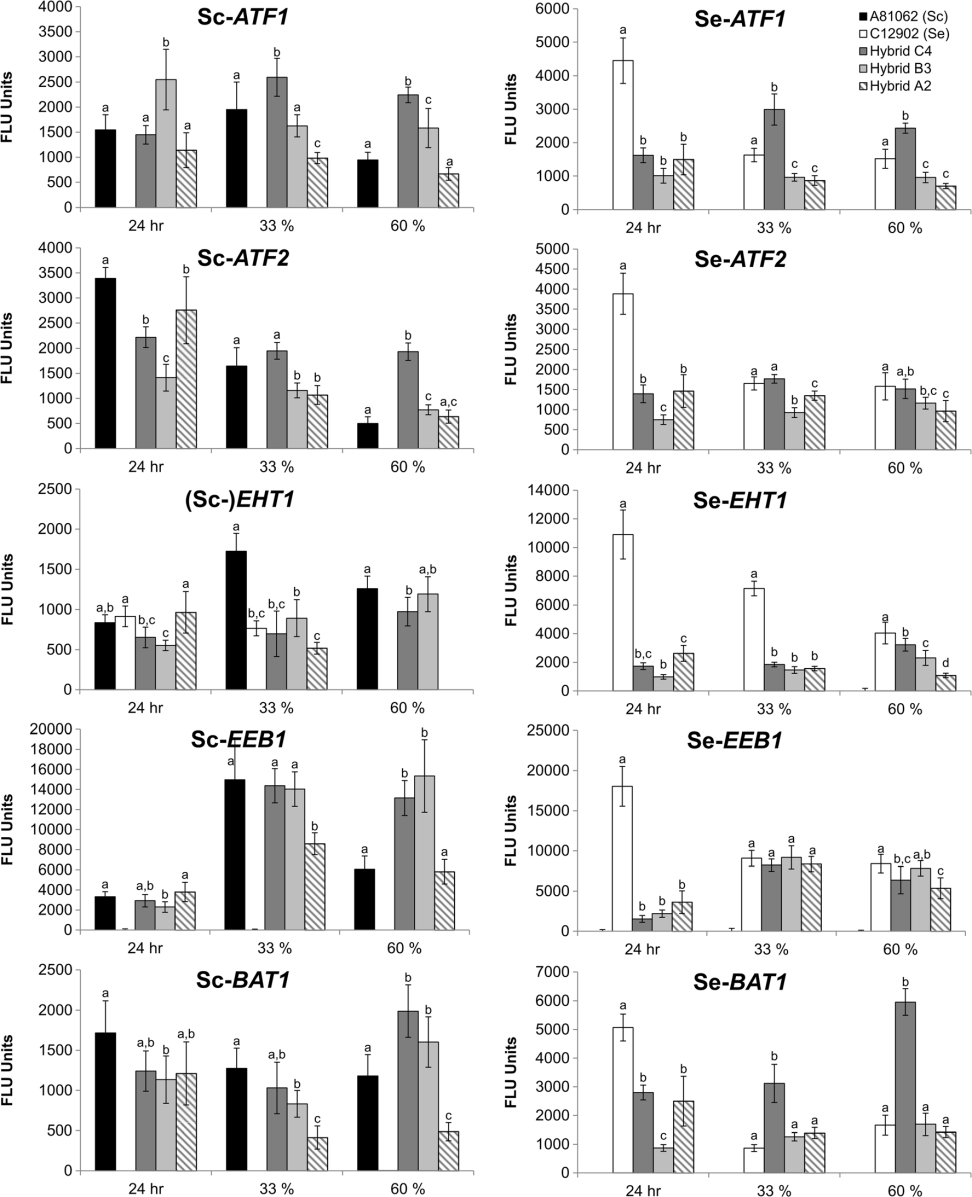
Transcription of *S. cerevisiae* (*Sc*) and *S. eubayanus* (*Se*) orthologues of genes responsible for ester formation during fermentation of the 15 °P wort with the hybrid and parent strains. Samples were taken at 24 h, 33 % attenuation, and 60 % attenuation. Values are means from two independent fermentations and *error bars* where visible represent the standard deviation. Values from the same sampling point with different *letters* (*a–d*) above the bars differ significantly (*p* < 0.05)

Higher or equal transcription levels of both Sc-*ATF1* and Se-*ATF1* were observed for the allotetraploid Hybrid C4 during active fermentation compared to the parent strains, with up to 2.5-fold and 2-fold differences, respectively (Fig. 6). The allotriploid Hybrid B3 also showed higher or equal transcription levels of Sc-*ATF1* compared to *S. cerevisiae* A81062, while the allodiploid Hybrid A2 showed up to 2-fold lower transcription levels. For Se-*ATF1*, both Hybrid B3 and Hybrid A2 showed approximately 2-fold lower transcription levels at 33 and 60 % attenuation compared to *S. eubayanus* C12902. Similar trends were observed for Sc-*ATF2* and Se-*ATF2*, where the overall highest levels of transcription among the hybrids were observed with Hybrid C4.

For Sc-*EHT1*, the highest transcription levels were observed for the *S. cerevisiae* parent strain, which showed a 2-fold difference at 33 % apparent attenuation compared to Hybrid B3 and Hybrid C4. A signal was obtained with the Sc-*EHT1* probe from the fermentations with the *S. eubayanus* parent strain, meaning that the probe was non-specific and cross-hybridized to something in the sample, possibly Se-*EHT1* mRNA. For Se-*EHT1*, the highest transcription levels were observed with the *S. eubayanus* parent strain throughout the fermentation. Towards the end of fermentation, the transcription level observed for Hybrid C4 was similar to the *S. eubayanus* parent. The transcription of Sc-*EEB1* was similar among the *S. cerevisiae* parent and all hybrid strains at 24 h and 33 % apparent attenuation, with the exception of Hybrid A2 at 33 % attenuation, where an approximately 2-fold lower transcription level was observed. Towards the end of fermentation, a 2.5-fold higher transcription level was observed for Hybrid C4 and Hybrid B3 relative to the allodiploid. For Se-*EEB1*, similar transcription levels were observed for all hybrid strains during active fermentation.

The transcription levels of Sc-*BAT1* followed similar patterns as Sc-*EEB1*, with little differences observed between the transcription levels of the *S. cerevisiae*, Hybrid C4, and Hybrid B3 in the beginning of fermentation, while transcription levels were around 1.5- to 2-fold higher in the hybrids towards the end of fermentation. The transcription level in the Hybrid A2 was up to 2.5-fold lower than the *S. cerevisiae* parent strain during active fermentation. For Se-*BAT1*, the transcription levels in the *S. eubayanus* parent, Hybrid B3, and Hybrid A2 were similar during active fermentation, but up to 4-fold higher in Hybrid C4.

In general, transcription levels tended to be equal or even higher in the hybrid strains that had inherited two copies of chromosomes from the parent. A Pearson product-moment correlation of 0.88 (*p* value lower than 0.0001) was obtained between the normalized average transcription levels of each orthologous gene in each strain (normalized to zero mean and unit variance) and the deduced gene copy numbers in Table 1. Linear regressions (adjusted *R*^2^ ranged from 0.68 to 0.89, and all *F* test *p* values were below 0.008; see Table S4 and S5 in Supplementary material) only revealed significant correlations between the maximum transcription levels of *ATF1* and *ATF2* and the concentrations 3-methylbutyl acetate and 2-phenylethyl acetate, as well as the maximum transcription levels of *EHT1* and *EEB1* and the concentrations of ethyl hexanoate (Table 3). For the concentrations of 3-methylbutyl acetate and 2-phenylethyl acetate, the expression of the *S. eubayanus-*derived orthologues of *ATF1* and *ATF2* had a larger positive influence than the *S. cerevisiae-*derived counterparts. For the concentrations of ethyl hexanoate, the expression of the *S. cerevisiae*-derived orthologues of *EHT1* and *EEB1* tended to have a larger positive influence compared to the *S. eubayanus-*derived orthologues.

**Table 3:** The *β* coefficients of the multiple linear regressions between maximum transcription levels and the beer concentrations of various aroma compounds

Gene	3-Methylbutyl acetate	2-Phenylethyl acetate	Gene	Ethyl hexanoate
Sc-*ATF1*	NS	NS	Sc-*EHT1*	NS
Se-*ATF1*	4.8 × 10^−4^	3.1 × 10^−4^	Se-*EHT1*	−3.8 × 10^−5^
Sc-*ATF2*	NS	NS	Sc-*EEB1*	1.6 × 10^−5^
Se-*ATF2*	5.8 × 10^−4^	3.7 × 10^−4^	Se-*EEB1*	NS

All coefficient values are significant (*p* < 0.05) and show a correlation between the transcription of that gene and the beer concentration of that aroma compound

*NS* not significant (*p* > 0.05)

## Discussion

While extensive research has been conducted on the development of brewing yeast through genetic engineering techniques in the past decades, the industrial use of genetically modified yeast is still not common as a result of regulations and public opinion (Cebollero et al. 2007; Stewart et al. 2013; Twardowski and Malyska 2015). Hence, there is a still a demand for developing and improving alternative, non-GM, strain-development techniques. One such technique is interspecific hybridization, which was used here to generate three lager yeast hybrids with varying DNA content. The purpose of this study was to compare the fermentation performance and aroma formation of these strains, and elucidate the relationship between the formation of aroma compounds and the expression of orthologous genes involved in their synthesis.

In the 15 °P wort, all three hybrid strains exhibited an apparent heterotic phenotype by outperforming both parent strains in fermentation rate and final alcohol yield. This is consistent with previous studies on yeast hybrids, where a heterotic effect is commonly observed (da Silva et al. 2015; Garcia Sanchez et al. 2012; Krogerus et al. 2015a; Mertens et al. 2015; Pérez-Través et al. 2015; Sato et al. 2002; Snoek et al. 2015). Furthermore, a growth assay (8–37 °C) also revealed a broader temperature range of growth for the hybrid strains compared to the parent strains (Fig. S13 in Supplementary material). The ability of the hybrid strains to ferment the wort efficiently at lower temperatures, which here was inherited from the *S. eubayanus* parent strain (Libkind et al. 2011), is essential for lager beer production. The hybrid vigor that was observed during both the 15 and 25 °P fermentations can partially be explained by the superior ability of the hybrid strains to take up and consume maltose and maltotriose from the wort. In brewing strains, the uptake of these sugars is carried out by various transmembrane transporters, such as Agt1, Malx1, and Mtt1 (Cousseau et al. 2013; Dietvorst et al. 2005; Vidgren et al. 2005, 2009). It has been shown that the activities of these transporters are dependent on temperature, origin, and membrane lipid composition (Guimarães et al. 2006; Vidgren et al. 2010, 2014), and that *S. eubayanus*-derived transporters in lager yeast tend to retain higher activities at lower temperatures compared to the *S. cerevisiae*-derived counterparts. Here, the hybrid strains, especially the allotetraploid Hybrid C4 and allotriploid Hybrid B3, were able to combine the efficient maltotriose use from the *S. cerevisiae* parent and the efficient maltose use from the *S. eubayanus* parent.

While all hybrid strains outperformed the parent strains in the 15 °P wort, it was only the allotriploid Hybrid B3 and allotetraploid Hybrid C4 that did so in the 25 °P very high gravity wort. These hybrids also showed a higher ethanol tolerance than the allodiploid Hybrid A2. Fermentation of very high gravity wort is limited by various environmental stresses, such as high osmotic pressure, high alcohol concentrations, and nutrient starvation (Blieck et al. 2007; Gibson et al. 2007; Gibson 2011). Our observations would suggest that polyploid hybrids possess increased stress tolerance, possibly as a result of increased gene dosage and positive selection of specific gene products, masking of deleterious recessive mutations, transcriptome changes, and even increased cell size (Schoenfelder and Fox 2015; Storchova 2014). Mechanisms affecting the ethanol and stress tolerance in yeast include the lipid composition of the plasma membrane (Henderson and Block 2014), intracellular trehalose concentrations (Bandara et al. 2009), and expression of general stress response genes (Sadeh et al. 2011). Studies have also shown that flocculating cells tend to be more tolerant towards ethanol, which is reflected in our results as well (Smukalla et al. 2008). It is unclear what factors contribute to the differences in ethanol and stress tolerance observed here between the hybrid and their parent strains, and these should be addressed in future work. The poor performance of the allodiploid Hybrid A2 in the 25 °P wort observed here is most likely coincidental, as previous studies have reported the generation of hybrid strains with superior ethanol production and tolerance through mass mating (Snoek et al. 2015; Zheng et al. 2011). On the other hand, Mertens et al. (2015) also observed poor fermentation performance with allodiploid yeast hybrids compared to allotriploid hybrids in pilot-scale wort fermentations. However, this may rather be associated with the performance of the parent strains. A more general observation could have been obtained by including a larger number of hybrids in the study.

Brewing yeast strain development is not only driven by a demand for faster and more tolerant brewing yeasts. The demand for brewing yeasts that produce novel and distinct flavor profiles has also increased in the past decade, as the beer industry has been driven by an increasing demand for craft and specialty beers that are rich in aroma (Aquilani et al. 2015). Our results suggest that it is not only possible to generate hybrid lager yeasts with higher aroma production compared to the parent strains but that the aroma profile of these hybrids can also be directed depending on the mating method and the DNA content and inheritance of the hybrids. Recent studies on other yeast hybrids have also revealed the possibility to either increase aroma production or achieve midparent values in hybrids (Bellon et al. 2011, 2013; da Silva et al. 2015; Gamero et al. 2013; Krogerus et al. 2015a; Mertens et al. 2015; Steensels et al. 2014a). Here, a positive correlation was observed both between transcription levels and gene copy numbers as well as the maximum transcription levels of several genes and the concentrations of corresponding aroma compounds in the beers. Hence, the increase in aroma production in the hybrid strains compared to the parents can be partly explained by the combined expression of both orthologous genes inherited from the parent strains. Previous studies on the expression of genes related to the synthesis of aroma compounds in newly formed interspecific yeast hybrids are limited. Nevertheless, studies on natural hybrids have revealed both that there is a positive correlation between the formation of aroma compounds and the expression level of several genes involved in their synthesis (e.g., *ATF1*, *ATF2*, and *BAT1*), and that orthologues of these genes in lager yeast are differentially transcribed during fermentation (He et al. 2014; Procopio et al. 2014; Saerens et al. 2008). Studies have shown that gene expression patterns in yeast hybrids are affected by environmental factors such as temperature (Li et al. 2012; Tirosh et al. 2009), which may also affect the contribution from the parental genomes in lager yeasts.

Aside from the observed link between ester concentrations and transcript levels, the results from transcriptional analysis also suggest that the functionality of the orthologous gene products differ. Four-fold higher concentrations of 3-methylbutyl acetate were observed in the 15 °P beer fermented with the *S. eubayanus* parent strain compared to *S. cerevisiae* parent strain, despite quite similar expression levels of the respective orthologous genes of *ATF1* and *ATF2*. Furthermore, similar concentrations of 3-methylbutyl acetate were measured in the beers produced with the *S. eubayanus* parent and the allotetraploid Hybrid C4, as well as the allotriploid Hybrid B3 and allodiploid Hybrid A2, despite the fact that higher concentrations of the precursor 3-methyl butanol were measured in the beers produced with the *S. eubayanus* parent and Hybrid A2. This suggests that the differences in ester concentrations were not limited by precursor availability. Surprisingly, relatively high concentrations of ethyl esters were observed in the beer fermented with the allodiploid Hybrid A2 despite it showing low expression levels of Sc-*EHT1*. These results, together with the multiple linear regression models, would suggest that the expression of Sc-*EEB1* has a stronger influence on the formation of ethyl esters, especially ethyl hexanoate, during fermentation, which is in agreement with previous studies (Saerens et al. 2008).

While the hybrid strains produced higher amounts of desirable aroma-active esters, they also produced higher concentrations of the undesirable off-flavor diacetyl. The post-fermentation removal of diacetyl can notably limit the production rate of lager beer (Krogerus and Gibson 2013b). Diacetyl formation is coupled with valine biosynthesis during fermentation, and the amount of diacetyl formed is linked to the activity of the *ILV2*- and *ILV6*-encoded acetohydroxyacid synthase enzyme and subunit. Recent studies have suggested a correlation between *ILV6* expression and diacetyl production (Duong et al. 2011; Gibson et al. 2015), while the sequencing of several industrial lager yeasts revealed higher copy numbers of *ILV* genes in strains producing more diacetyl (Van den Broek et al. 2015). The expression of *ILV2* and *ILV6* genes was not monitored during this study, but the sequencing data suggest they were present in higher copy numbers in the allotriploid and allotetraploid hybrid strains (data not shown). Environmental factors also affect diacetyl formation and removal to a large extent. For example, a low wort pH increases α-acetolactate decarboxylation and diacetyl removal rates, which here explain the more rapid diacetyl removal that was observed for the allodiploid Hybrid A2 compared to the other hybrid strains (Krogerus et al. 2015b).

The variation that was observed between the hybrid strains in fermentation characteristics and aroma profiles highlights several benefits and drawbacks of both the hybrid generation methods, rare mating and spore mating. The hybrids produced through rare mating, Hybrid C4 and Hybrid B3, outperformed the parent strains and the allodiploid Hybrid A2 during fermentation and, in the case of the allotetraploid Hybrid C4, produced beer with the highest concentration of flavor-active esters. While they also showed higher ethanol tolerances, they had lower viabilities after fermentation. It has also been shown that the genetic stability of hybrids produced with rare mating is lower than that of spore-to-spore hybrids (Pérez-Través et al. 2012; Kunicka-Styczynska and Rajkowska 2011), which here was evident by the apparent chromosome losses in the allotetraploid Hybrid C4 (a loss of one *S. eubayanus*-derived chromosome X). Hybrids B3 and C4 also contained only a single copy of the *S. cerevisiae*-derived chromosome III, containing the mating type locus. However, it is likely that this loss occurred prior to the hybridization, and this loss of heterozygosity allowed rare mating to occur (Hiraoka et al. 2000). Similar losses of chromosome III can also be observed in industrial lager strains (Walther et al. 2014; Van den Broek et al. 2015). Genetic instability could be exploited for further strain development through adaptive evolution, as it was recently shown that polyploid yeast undergo faster adaptation (Selmecki et al. 2015). Further research should be carried out in order to investigate the long-term stability of these hybrids strains, e.g., through serial repitching and the monitoring of genome stabilization and chromosomal rearrangements.

An aspect that considerably limits the applicability of spore-to-spore mating is the fact that sporulation is often poor in industrial brewing yeasts (Bilinski et al. 1986). However, it is expected that the diversity among hybrids generated by spore-to-spore mating should be greater than those generated through rare mating, as a result of meiotic recombination during spore formation (Marullo et al. 2004; Neiman 2011). This may result in the diploid hybrids losing phenotypic traits from the parent strains (Pérez-Través et al. 2015), as was observed here, e.g., for flocculation, where a much lower flocculation ability was observed for the allodiploid Hybrid A2 compared to the Hybrid B3 and Hybrid C4. However, this can also be used beneficially to eliminate unwanted phenotypic traits, such as excessive flocculation and production of phenolic off-flavors (Russell et al. 1983). This is relevant to the present study as well since a clear phenolic clove-like aroma, caused by the presence of 4-vinylguaiacol (Coghe et al. 2004), was detected in all the beers. This aroma is typically unwanted in lager beer, and therefore future attempts should be made to remove this characteristic and increase the industrial applicability of these new hybrid lager strains. As allodiploid hybrids produced by spore-to-spore mating are susceptible to genetic segregation through meiosis, especially where crosses involve heterozygous parent strains, future studies could assess genetic and phenotypic variation that exists among such hybrids. Homozygous parent strains could be created to reduce the effect of recombination and could act as control strains in such studies.

In conclusion, the results of this study show that interspecific hybridization is a useful non-GM tool for improving and developing brewing yeast, and that the physiological properties of these newly generated hybrids can be controlled to some extent through their ploidy and subgenome inheritance. The hybrids not only outperformed the parent strains in relation to fermentation rate but also produced beer with higher concentrations of certain flavor-active esters. Transcriptional analysis revealed that the increased formation of esters in the hybrid strains could be partly explained by the combined, and sometimes even increased, gene transcription levels of orthologous genes inherited from both parent strains. Further research combining interspecific hybridization and adaptive evolution could yield additional powerful tools for the creation of bespoke lager yeast.

## Electronic supplementary material

ESM 1 (PDF 2.44 mb)

## References

[CR1] Abyzov A, Urban A, Snyder M, Gerstein M (2011). CNVnator: an approach to discover, genotype, and characterize typical and atypical CNVs from family and population genome sequencing. Genome Res.

[CR2] Aquilani B, Laureti T, Poponi S, Secondi L (2015). Beer choice and consumption determinants when craft beers are tasted: an exploratory study of consumer preferences. Food Qual Prefer.

[CR3] Baker E, Wang B, Bellora N, Peris D, Hulfachor A, Koshalek J, Adams M, Libkind D, Hittinger C (2015). The genome sequence of *Saccharomyces eubayanus* and the domestication of lager-brewing yeasts. Mol Biol Evol.

[CR4] Bandara A, Fraser S, Chambers P, Stanley G (2009). Trehalose promotes the survival of *Saccharomyces cerevisiae* during lethal ethanol stress, but does not influence growth under sublethal ethanol stress. FEMS Yeast Res.

[CR5] Bellon J, Eglinton J, Siebert T, Pollnitz A, Rose L, de Barros LM, Chambers P (2011). Newly generated interspecific wine yeast hybrids introduce flavour and aroma diversity to wines. Appl Microbiol Biotechnol.

[CR6] Bellon J, Schmid F, Capone D, Dunn B, Chambers P (2013). Introducing a new breed of wine yeast: interspecific hybridisation between a commercial *Saccharomyces cerevisiae* wine yeast and *Saccharomyces mikatae*. PLoS ONE.

[CR7] Bilinski C, Russell I, Stewart G (1986). Analysis of sporulation in brewer’s yeast: induction of tetrad formation. J Inst Brew.

[CR8] Blieck L, Toye G, Dumortier F, Verstrepen K, Delvaux F, Thevelein J, Van Dijck P (2007). Isolation and characterization of brewer’s yeast variants with improved fermentation performance under high-gravity conditions. Appl Environ Microbiol.

[CR9] Boeke J, Trueheart J, Natsoulis G, Fink G (1987). 5-Fluoroorotic acid as a selective agent in yeast molecular genetics. Methods Enzymol.

[CR10] Boetzer M, Pirovana W (2012). Toward almost closed genomes with GapFiller. Genome Biol.

[CR11] Boetzer M, Pirovano W (2014). SSPACE-LongRead: scaffolding bacterial draft genomes using long read sequence information. BMC Bioinf.

[CR12] Bolat I, Romagnoli G, Zhu F, Pronk J, Daran J (2013). Functional analysis and transcriptional regulation of two orthologs of *ARO10*, encoding broad-substrate-specificity 2-oxo-acid decarboxylases, in the brewing yeast *Saccharomyces pastorianus* CBS1483. FEMS Yeast Res.

[CR13] Cebollero E, Gonzalez-Ramos D, Tabera L, Gonzalez R (2007). Transgenic wine yeast technology comes of age: is it time for transgenic wine?. Biotechnol Lett.

[CR14] Chaisson M, Tesler G (2012). Mapping single molecule sequencing reads using basic local alignment with successive refinement (BLASR): application and theory. BMC Bioinf.

[CR15] Chen Z (2007). Genetic and epigenetic mechanisms for gene expression and phenotypic variation in plant polyploids. Annu Rev Plant Biol.

[CR16] Chen Z (2013). Genomic and epigenetic insights into the molecular bases of heterosis. Nat Rev Genet.

[CR17] Chiang C, Layer R, Faust G, Lindberg M, Rose D, Garrison E, Marth G, Quinlan A, Hall I (2015). SpeedSeq: ultra-fast personal genome analysis and interpretation. Nat Methods.

[CR18] Coghe S, Benoot K, Delvaux F, Vanderhaegen B, Delvaux FR (2004). Ferulic acid release and 4-vinylguaiacol formation during brewing and fermentation: indication for feruloyl esterase activity in *Saccharomyces cerevisiae*. J Agric Food Chem.

[CR19] Cousseau F, Alves S, Trichez D, Stambuk B (2013). Characterization of maltotriose transporters from the *Saccharomyces eubayanus* subgenome of the hybrid *Saccharomyces pastorianus* lager brewing yeast strain Weihenstephan 34/70. Lett Appl Microbiol.

[CR20] D’Hautcourt O, Smart K (1999). Measurement of brewing yeast flocculation. J Am Soc Brew Chem.

[CR21] da Silva T, Albertin W, Dillmann C, Bely M, la Guerche S, Giraud C, Huet S, Sicard D, Masneuf-Pomarede I, de Vienne D, Marullo P (2015). Hybridization within *Saccharomyces* genus results in homoeostasis and phenotypic novelty in winemaking conditions. PLoS ONE.

[CR22] Dietvorst J, Londesborough J, Steensma HY (2005). Maltotriose utilization by lager yeast strains: MTT1 encodes a maltotriose transporter. Yeast.

[CR23] Dunn B, Sherlock G (2008). Reconstruction of the genome origins and evolution of the hybrid lager yeast *Saccharomyces pastorianus*. Genome Res.

[CR24] Duong C, Strack L, Futschik M, Katou Y, Nakao Y, Fujimura T, Shirahige K, Kodama Y, Nevoigt E (2011). Identification of Sc-type *ILV6* as a target to reduce diacetyl formation in lager brewers’ yeast. Metab Eng.

[CR25] Engel S, Dietrich F, Fisk D, Binkley G, Balakrishnan R, Costanzo M, Dwight S, Hitz B, Karra K, Nash R, Weng S, Wong E, Lloyd P, Skrzypek M, Miyasato S, Simison M, Cherry J (2013). The reference genome sequence of *Saccharomyces cerevisiae*: then and now. G3.

[CR26] European Brewery Convention (2004). Analytica-EBC.

[CR27] Fu D, Xiao M, Hayward A, Jiang G, Zhu L, Zhou Q, Li J, Zhang M (2015). What is crop heterosis: new insights into an old topic. J Appl Genet.

[CR28] Gamero A, Tronchoni J, Querol A, Belloch C (2013). Production of aroma compounds by cryotolerant *Saccharomyces* species and hybrids at low and moderate fermentation temperatures. J Appl Microbiol.

[CR29] Garcia Sanchez R, Solodovnikova N, Wendland J (2012). Breeding of lager yeast with *Saccharomyces cerevisiae* improves stress resistance and fermentation performance. Yeast.

[CR30] García-Alcalde F, Okonechnikov K, Carbonell J, Cruz L, Götz S, Tarazona S, Dopazo J, Meyer T, Conesa A (2012). Qualimap: evaluating next-generation sequencing alignment data. Bioinformatics.

[CR31] Garrison E, Marth G (2012) Haplotype-based variant detection from short-read sequencing. arXiv:1207.3907

[CR32] Gibson B (2011). 125th anniversary review: improvement of higher gravity brewery fermentation via wort enrichment and supplementation. J Inst Brew.

[CR33] Gibson B, Liti G (2015). *Saccharomyces pastorianus*: genomic insights inspiring innovation for industry. Yeast.

[CR34] Gibson B, Lawrence S, Leclaire J, Powell C, Smart K (2007). Yeast responses to stresses associated with industrial brewery handling. FEMS Microbiol Rev.

[CR35] Gibson B, Storgårds E, Krogerus K, Vidgren V (2013). Comparative physiology and fermentation performance of Saaz and Frohberg lager yeast strains and the parental species *Saccharomyces eubayanus*. Yeast.

[CR36] Gibson B, Londesborough J, Rautio K, Mattinen L, Vidgren V (2013). Transcription of α-glucoside transport and metabolism genes in the hybrid brewing yeast *Saccharomyces pastorianus* with respect to gene provenance and fermentation temperature. J Inst Brew.

[CR37] Gibson B, Krogerus K, Ekberg J, Monroux A, Mattinen L, Rautio J, Vidgren V (2015). Variation in α-acetolactate production within the hybrid lager yeast group *Saccharomyces pastorianus* and the affirmation of the central role of the *ILV6* gene. Yeast.

[CR38] Gueldener U, Heinisch J, Koehler G, Voss D, Hegemann J (2002). A second set of *loxP* marker cassettes for Cre-mediated multiple gene knockouts in budding yeast. Nucleic Acids Res.

[CR39] Guimarães P, Virtanen H, Londesborough J (2006). Direct evidence that maltose transport activity is affected by the lipid composition of brewer’s yeast. J Inst Brew.

[CR40] Haas B, Salzberg S, Zhu W, Pertea M, Allen J, Orvis J, White O, Buell C, Wortman J (2008). Automated eukaryotic gene structure annotation using EVidenceModeler and the program to assemble spliced alignments. Genome Biol.

[CR41] Haase S, Reed S (2002). Improved flow cytometric analysis of the budding yeast cell cycle. Cell Cycle.

[CR42] He Y, Dong J, Yin H, Chen P, Lin H, Chen L (2014). Monitoring of the production of flavour compounds by analysis of the gene transcription involved in higher alcohol and ester formation by the brewer’s yeast *Saccharomyces pastorianus* using a multiplex RT-qPCR assay. J Inst Brew.

[CR43] Hebly M, Brickwedde A, Bolat I, Driessen M, de Hulster E, van den Broek M, Pronk J, Geertman J, Daran J, Daran-Lapujade P (2015). *S. cerevisiae* × *S. eubayanus* interspecific hybrid, best of both worlds and beyond. FEMS Yeast Res.

[CR44] Henderson C, Block D (2014). Examining the role of membrane lipid composition in determining the ethanol tolerance of *Saccharomyces cerevisiae*. Appl Environ Microbiol.

[CR45] Hiralal L, Olaniran A, Pillay B (2014). Aroma-active ester profile of ale beer produced under different fermentation and nutritional conditions. J Biosci Bioeng.

[CR46] Hiraoka M, Watanabe K, Umezu K, Maki H (2000). Spontaneous loss of heterozygosity in diploid *Saccharomyces cerevisiae* cells. Genetics.

[CR47] Holt C, Yandell M (2011). MAKER2: an annotation pipeline and genome-database management tool for second-generation genome projects. BMC Bioinf.

[CR48] Horinouchi T, Yoshikawa K, Kawaide R, Furusawa C, Nakao Y, Hirasawa T, Shimizu H (2010). Genome-wide expression analysis of *Saccharomyces pastorianus* orthologous genes using oligonucleotide microarrays. J Biosci Bioeng.

[CR49] Jiang H, Lei R, Ding S, Zhu S (2014). Skewer: a fast and accurate adapter trimmer for next-generation sequencing paired-end reads. BMC Bioinf.

[CR50] Klambauer G, Schwarzbauer K, Mayr A, Clevert D, Mitterecker A, Bodenhofer U, Hochreiter S (2012). Cn.MOPS: mixture of Poissons for discovering copy number variations in next-generation sequencing data with a low false discovery rate. Nucleic Acids Res.

[CR51] Kolmogorov M, Raney B, Paten B, Pham S (2014). Ragout—a reference-assisted assembly tool for bacterial genomes. Bioinformatics.

[CR52] Krogerus K, Gibson B (2013). Influence of valine and other amino acids on total diacetyl and 2,3-pentanedione levels during fermentation of brewer’s wort. Appl Microbiol Biotechnol.

[CR53] Krogerus K, Gibson B (2013). 125th anniversary review: diacetyl and its control during brewery fermentation. J Inst Brew.

[CR54] Krogerus K, Magalhães F, Vidgren V, Gibson B (2015). New lager yeast strains generated by interspecific hybridization. J Ind Microbiol Biotechnol.

[CR55] Krogerus K, Gibson B, Hytönen E (2015). An improved model for prediction of wort fermentation progress and total diacetyl profile. J Am Soc Brew Chem.

[CR56] Kunicka-Styczynska A, Rajkowska K (2011). Physiological and genetic stability of hybrids of industrial wine yeasts *Saccharomyces* sensu stricto complex. J Appl Microbiol.

[CR57] Lawrence M, Huber W, Pagès H, Aboyoun O, Carlson M, Gentleman R, Morgan M, Carey V (2013). Software for computing and annotating genomic ranges. PLoS Comput Biol.

[CR58] Li H, Handsaker B, Wysoker A, Fennell T, Ruan J, Homer N, Marth G, Abecasis G, Durbin R, 1000 Genome Project Data Processing Subgroup (2009). The sequence alignment/map format and SAMtools. Bioinformatics.

[CR59] Li C, Tzeng J, Huang-Mo S (2012). Effects of *cis* and *trans* regulatory variations on the expression divergence of heat shock response genes between yeast strains. Gene.

[CR60] Libkind D, Hittinger C, Valerio E, Goncalves C, Dover J, Johnston M, Goncalves P, Sampaio J (2011). Microbe domestication and the identification of the wild genetic stock of lager-brewing yeast. Proc Natl Acad Sci U S A.

[CR61] Lilly M, Bauer F, Styger G, Lambrechts M, Pretorius I (2006). The effect of increased branched-chain amino acid transaminase activity in yeast on the production of higher alcohols and on the flavour profiles of wine and distillates. FEMS Yeast Res.

[CR62] Lippman Z, Zamir D (2007). Heterosis: revisiting the magic. Trends Genet.

[CR63] Liti G, Peruffo A, James S, Roberts I, Louis E (2005). Inferences of evolutionary relationships from a population survey of LTR-retrotransposons and telomeric-associated sequences in the *Saccharomyces sensu stricto* complex. Yeast.

[CR64] Marullo P, Bely M, Masneuf-Pomarede I, Aigle M, Dubourdieu D (2004). Inheritable nature of enological quantitative traits is demonstrated by meiotic segregation of industrial wine yeast strains. FEMS Yeast Res.

[CR65] Meilgaard M (1982). Prediction of flavor differences between beers from their chemical composition. J Agric Food Chem.

[CR66] Mertens S, Steensels J, Saels V, de Rouck G, Aerts G, Verstrepen K (2015). A large set of newly created interspecific yeast hybrids increases aromatic diversity in lager beers. Appl Environ Microbiol.

[CR67] Morgan M, Pagès H, Obenchain V, Hayden N (2010) Rsamtools: binary alignment (BAM), FASTA, variant call (BCF), and tabix file import. R package version 1.22.0, http://bioconductor.org/packages/release/bioc/html/Rsamtools.html

[CR68] Muir A, Harrison E, Wheals A (2011). A multiplex set of species-specific primers for rapid identification of members of the genus *Saccharomyces*. FEMS Yeast Res.

[CR69] Nakao Y, Kanamori T, Itoh T, Kodama Y, Rainieri S, Nakamura N, Shimonaga T, Hattori M, Ashikari T (2009). Genome sequence of the lager brewing yeast, an interspecies hybrid. DNA Res.

[CR70] Neiman A (2011). Sporulation in the budding yeast *Saccharomyces cerevisiae*. Genetics.

[CR71] Otto T, Dillon G, Degrave W, Berriman M (2011). RATT: rapid annotation transfer tool. Nucleic Acids Res.

[CR72] Pengelly R, Wheals A (2013). Rapid identification of *Saccharomyces eubayanus* and its hybrids. FEMS Yeast Res.

[CR73] Pérez-Través L, Lopes C, Barrio E, Querol A (2012). Evaluation of different genetic procedures for the generation of artificial hybrids in *Saccharomyces* genus for winemaking. Int J Food Microbiol.

[CR74] Pérez-Través L, Lopes C, González R, Barrio E, Querol A (2015). Physiological and genomic characterisation of *Saccharomyces cerevisiae* hybrids with improved fermentation performance and mannoprotein release capacity. Int J Food Microbiol.

[CR75] Pfaffl M (2001). A new mathematical model for relative quantification in real-time RT-PCR. Nucleic Acids Res.

[CR76] Pham T, Wimalasena T, Box W, Koivuranta K, Storgårds E, Smart K, Gibson B (2011). Evaluation of ITS PCR and RFLP for differentiation and identification of brewing yeast and brewery ‘wild’ yeast contaminants. J Inst Brew.

[CR77] Pires E, Teixeira J, Branyik T, Vicente A (2014). Yeast: the soul of beer’s aroma—a review of flavour-active esters and higher alcohols produced by the brewing yeast. Appl Microbiol Biotechnol.

[CR78] Plech M, de Visser J, Korona R (2014). Heterosis is prevalent among domesticated but not wild strains of *Saccharomyces cerevisiae*. G3.

[CR79] Procopio S, Brunner M, Becker T (2014). Differential transcribed yeast genes involved in flavour formation and its associated amino acid metabolism during brewery fermentation. Eur Food Res Technol.

[CR80] Proux-Wéra E, Armisén D, Byrne K, Wolfe K (2012). A pipeline for automated annotation of yeast genome sequences by a conserved-synteny approach. BMC Bioinf.

[CR81] Rautio J, Huuskonen A, Vuokko H, Vidgren V, Londesborough J (2007). Monitoring yeast physiology during very high gravity wort fermentations by frequent analysis of gene expression. Yeast.

[CR82] Russell I, Hancock I, Stewart G (1983). Construction of dextrin fermentative yeast strains that do not produce phenolic off-flavours in beer. J Am Soc Brew Chem.

[CR83] Sadeh A, Movshovich N, Volokh M, Gheber L, Aharoni A (2011). Fine-tuning of the Msn2/4-mediated yeast stress responses as revealed by systematic deletion of Msn2/4 partners. Mol Biol Cell.

[CR84] Saerens S, Verstrepen K, Van Laere S, Voet A, Van Dijck P, Delvaux FR, Thevelein J (2006). The *Saccharomyces cerevisiae* EHT1 and EEB1 genes encode novel enzymes with medium-chain fatty acid ethyl ester synthesis and hydrolysis capacity. J Biol Chem.

[CR85] Saerens S, Verbelen P, Vanbeneden N, Thevelein J, Delvaux FR (2008). Monitoring the influence of high-gravity brewing and fermentation temperature on flavour formation by analysis of gene expression levels in brewing yeast. Appl Microbiol Biotechnol.

[CR86] Sato M, Kishimoto M, Watari J, Takashio M (2002). Breeding of brewer’s yeast by hybridization between a top-fermenting yeast *Saccharomyces cerevisiae* and a cryophilic yeast *Saccharomyces bayanus*. J Biosci Bioeng.

[CR87] Scannell D, Zill O, Rokas A, Payen C, Dunham M, Eisen M, Rine J, Johnston M, Hittinger C (2011). The awesome power of yeast evolutionary genetics: new genome sequences and strain resources for the *Saccharomyces sensu stricto* genus. G3.

[CR88] Schoenfelder K, Fox D (2015). The expanding implications of polyploidy. J Cell Biol.

[CR89] Selmecki A, Maruvka Y, Richmond P, Guillet M, Shoresh N, Sorenson A, De S, Kishony R, Michor F, Dowell R, Pellman D (2015). Polyploidy can drive rapid adaptation in yeast. Nature.

[CR90] Shapira R, Levy T, Shaked S, Fridman E, David L (2014). Extensive heterosis in growth of yeast hybrids is explained by a combination of genetic models. Heredity.

[CR91] Smukalla S, Caldara M, Pochet N, Beauvais A, Guadagnini S, Yan C, Vinces M, Jansen A, Prevost M, Latgé J, Fink G, Foster K, Verstrepen K (2008). *FLO1* is a variable green beard gene that drives biofilm-like cooperation in budding yeast. Cell.

[CR92] Snoek T, Picca Nicolino M, Van den Bremt S, Mertens S, Saels V, Verplaetse A, Steensels J, Verstrepen K (2015). Large-scale robot-assisted genome shuffling yields industrial *Saccharomyces cerevisiae* yeasts with increased ethanol tolerance. Biotechnol Biofuels.

[CR93] Steensels J, Meersman E, Snoek T, Saels V, Verstrepen K (2014). Large-scale selection and breeding to generate industrial yeasts with superior aroma production. Appl Environ Microbiol.

[CR94] Steensels J, Snoek T, Meersman E, Picca Nicolino M, Voordeckers K, Verstrepen K (2014). Improving industrial yeast strains: exploiting natural and artificial diversity. FEMS Microbiol Rev.

[CR95] Stewart G, Hill A, Russell I (2013). 125th anniversary review: developments in brewing and distilling yeast strains. J Inst Brew.

[CR96] Storchova Z (2014). Ploidy changes and genome stability in yeast. Yeast.

[CR97] Stribny J, Gamero A, Pérez-Torrado R, Querol A (2015). *Saccharomyces kudriavzevii* and *Saccharomyces uvarum* differ from *Saccharomyces cerevisiae* during the production of aroma-active higher alcohols and acetate esters using their amino acidic precursors. Int J Food Microbiol.

[CR98] Tirosh I, Reikhav S, Levy A, Barkai N (2009). A yeast hybrid provides insight into the evolution of gene expression regulation. Science.

[CR99] Twardowski T, Malyska A (2015). Uninformed and disinformed society and the GMO market. Trends Biotechnol.

[CR100] Van den Broek M, Bolat I, Nijkamp J, Ramos E, Luttik M, Koopman F, Geertman J, de Ridder D, Pronk J, Daran J (2015). Chromosomal copy number variation in *Saccharomyces pastorianus* evidence for extensive genome dynamics in industrial lager brewing strains. Appl Environ Microbiol.

[CR101] Verstrepen K, Van Laere S, Vanderhaegen B, Derdelinckx G, Dufour J, Pretorius I, Winderickx J, Thevelein J, Delvaux FR (2003). Expression levels of the yeast alcohol acetyltransferase genes *ATF1*, *Lg-ATF1*, and *ATF2* control the formation of a broad range of volatile esters. Appl Environ Microbiol.

[CR102] Vidgren V, Ruohonen L, Londesborough J (2005). Characterization and functional analysis of the *MAL* and *MPH* loci for maltose utilization in some ale and lager yeast strains. Appl Environ Microbiol.

[CR103] Vidgren V, Huuskonen A, Virtanen H, Ruohonen L, Londesborough J (2009). Improved fermentation performance of a lager yeast after repair of its *AGT1* maltose and maltotriose transporter genes. Appl Environ Microbiol.

[CR104] Vidgren V, Multanen J, Ruohonen L, Londesborough J (2010). The temperature dependence of maltose transport in ale and lager strains of brewer’s yeast. FEMS Yeast Res.

[CR105] Vidgren V, Viljanen K, Mattinen L, Rautio J, Londesborough J (2014). Three Agt1 transporters from brewer’s yeasts exhibit different temperature dependencies for maltose transport over the range of brewery temperatures (0–20 °C). FEMS Yeast Res.

[CR106] Walther A, Hesselbart A, Wendland J (2014). Genome sequence of *Saccharomyces carlsbergensis*, the world’s first pure culture lager yeast. G3.

[CR107] Yamada R, Tanaka T, Ogino C, Kondo A (2010). Gene copy number and polyploidy on products formation in yeast. Appl Microbiol Biotechnol.

[CR108] Yao H, Dogra Gray A, Auger D, Birchler J (2013). Genomic dosage effects on heterosis in triploid maize. Proc Natl Acad Sci U S A.

[CR109] Yoshioka K, Hashimoto N (1981). Ester formation by alcohol acetyltransferase from brewer’s yeast. Agric Biol Chem.

[CR110] Zaret K, Sherman F (1985). Alpha-aminoadipate as a primary nitrogen source for *Saccharomyces cerevisiae* mutants. J Bacteriol.

[CR111] Zhang C, Liu Y, Qi Y, Zhang J, Dai L, Lin X, Ziao D (2013). Increased esters and decreased higher alcohols production by engineered brewer’s yeast strains. Eur Food Res Technol.

[CR112] Zheng D, Wu X, Tao X, Wang P, Li P, Chi X, Li Y, Yan Q, Zhao Y (2011). Screening and construction of *Saccharomyces cerevisiae* strains with improved multi-tolerance and bioethanol fermentation performance. Bioresour Technol.

