# Supplementary material for: Ploidy influences the functional attributes of de novo lager yeast hybrids
Source: Appl Microbiol Biotechnol. 2016 May 17;100:7203–22. doi: 10.1007/s00253-016-7588-3 (PMC4947488; doi:10.1007/s00253-016-7588-3)
Supplement: Supplementary file 1 — (PDF 2.44 mb) [file 253_2016_7588_MOESM1_ESM.pdf]

Supplementary material

Manuscript title: Ploidy influences the functional attributes of *de novo* lager yeast hybrids

Applied Microbiology and Biotechnology

Kristoffer Krogerus*, Mikko Arvas, Matteo De Chiara, Frederico Magalhães, Laura Mattinen, Merja Oja, Jia-Xing Yue, Virve Vidgren, Gianni Liti, Brian Gibson

VTT Technical Research Centre of Finland

Tietotie 2

P.O. Box 1000

FI-02044 VTT, Espoo

Finland

*Email: kristoffer.krogerus@aalto.fi

Table S1 – Oligonucleotide probes used for yeast transcriptional analysis with the TRAC assay.

Probe	Probe ID	Probe sequence
Sc-*ATF1*	4150	ACCCGAAAGAAGTGACTTCGGAATAAACAAGTACGGT
Se-*ATF1*	4143	TGTTGAATTTGCTTATCCATGGAGTGAAGTCAACAAACCC
Sc-*ATF2*	4132	AAGAGTTGGCAAGAAGGCTGTCTATATTCTT
Se-*ATF2*	4155	TCTCCTCGATCGTAGAGAGTAAAGCAGTCAAATCCTG
Sc-*BAT1*	4096	AAGACGTACGGCTTTGAAACCAGTCTTATAATAAGGACCGACTGGA
Se-*BAT1*	3897	TCCTCTTTTAGCAGCTTGCAATTGAGGTAAGATACATGGA
Sc-*EEB1*	4144	TAAAACTAACTCATCAAAGCTACCCAAGAACTCGGCAGCTTGC
Se-*EEB1*	4147	CAGCTCCAAAGGAAGTCCCTACAGCGTATAATTTCCTATGAGGAAATCT
Sc-*EHT1*	4145	ACAACAGGATCATCCCTGGAATTGATAACAAGGGTAGGAACCT
Se-*EHT1*	4133	ACAGACACCGCCATCCGAGAACTCGACGATTTCC

Table S2 – Primers used for estimating the relative copy numbers of *ATF1*, *ATF2* and *EEB1* by quantitative PCR.

Primer	Sequence (5' → 3')	Amplicon size (bp)
*BLE1*_FW	CCATCACGAGATTTCGATTCC	116
*BLE1*_RV	GAAGAACTCCAGCTTGAGATCC	
Sc-*ATF1*_FW	CACATTAGCTTTGAGGGAAATCTG	183
Sc-*ATF1*_RV	TTCATTGAGAACCACACCAC	
Se-*ATF1*_FW	GGTTTGTTGACTTCACTCCA	278
Se-*ATF1*_RV	GATACTTGTGTTCGGTCTCCT	
Sc-*ATF2*_FW	AAACCTCCAGCGAATCTACAC	204
Sc-*ATF2*_RV	AACAGCTTATCCAGTCTATACAGG	
Se-*ATF2*_FW	AGAGACAACTGAGACTTGCC	233
Se-*ATF2*_RV	GTTCCGAAACTTTGCTGATGAC	
Sc-*EEB1*_FW	GATTCTAAGCGCCAAATTCCTC	231
Sc-*EEB1*_RV	GCTCCCATTCAGGCATAACC	
Se-*EEB1*_FW	GGACGAGATACCTAACCTCAG	221
Se-*EEB1*_RV	TGAAGAGACTACGGGTTGTG	

Table S3 – The copy numbers of *ATF1*, *ATF2* and *EEB1* in the hybrid strains relative to the parent strains as determined by quantitative PCR.

**Strain**	Sc-*ATF1*	Sc-*ATF2*	Sc-*EEB1*	Se-*ATF1*	Se-*ATF2*	Se-*EEB1*
Hybrid A2	1.1 (0.3)	1.0 (0.2)	0.9 (0.2)	1.2 (0.1)	0.9 (0.2)	0.9 (0.1)
Hybrid B3	2.1 (0.3)	2.0 (0.3)	1.9 (0.4)	1.2 (0.3)	1.2 (0.2)	1.1 (0.2)
Hybrid C4	2.2 (0.4)	2.2 (0.4)	2.0 (0.4)	1.9 (0.3)	2.0 (0.1)	1.8 (0.3)

Values are means from four independent replicates (standard deviation in parenthesis).

Table S4 – The parameters of the linear regressions between the maximum transcription levels of *ATF1* and *ATF2* genes and the beer concentrations of 3-methylbutyl acetate, 2-phenylethyl acetate and ethyl acetate.

**Gene**	**3-Methylbutyl acetate**						**2-Phenylethyl acetate**						**Ethyl acetate**					
	**β coefficient**	**Standard Error of β**	***p*-value of β**	**R^2**	**Adjusted R^2**	***F*-test *p*-value**	**β coefficient**	**Standard Error of β**	***p*-value of β**	**R^2**	**Adjusted R^2**	***F*-test *p*-value**	**β coefficient**	**Standard Error of β**	***p*-value of β**	**R^2**	**Adjusted R^2**	***F*-test *p*-value**
Sc-*ATF1*	$1.2 \cdot 10^{-4}$	$1.1 \cdot 10^{-4}$	0.291	0.89	0.86	$4 \cdot 10^{-4}$	$-6.2 \cdot 10^{-5}$	$6.8 \cdot 10^{-5}$	0.391	0.92	0.89	$2 \cdot 10^{-4}$	$-2.8 \cdot 10^{-3}$	$4.8 \cdot 10^{-3}$	0.582	0.09	-0.17	0.72
Se-*ATF1*	**$4.8 \cdot 10^{-4}$**	$6.9 \cdot 10^{-5}$	$2 \cdot 10^{-4}$				**$3.1 \cdot 10^{-4}$**	$4.5 \cdot 10^{-5}$	$2 \cdot 10^{-4}$				$-2.5 \cdot 10^{-3}$	$3.1 \cdot 10^{-3}$	0.443			
Sc-*ATF2*	$9.8 \cdot 10^{-5}$	$2.1 \cdot 10^{-4}$	0.656	0.75	0.68	0.008	$-2.6 \cdot 10^{-5}$	$1.3 \cdot 10^{-4}$	0.844	0.83	0.78	0.002	$5.5 \cdot 10^{-3}$	$6.2 \cdot 10^{-3}$	0.402	0.11	-0.15	0.67
Se-*ATF2*	**$5.9 \cdot 10^{-4}$**	$2.0 \cdot 10^{-4}$	0.024				**$3.7 \cdot 10^{-4}$**	$1.2 \cdot 10^{-4}$	0.019				$3.7 \cdot 10^{-3}$	$5.9 \cdot 10^{-3}$	0.548			

A **bolded** β coefficient suggests a significant ($p < 0.05$) correlation between the transcription of that gene and that aroma compound.

Table S5 – The parameters of the linear regressions between the maximum transcription levels of *EHT1* and *EEB1* genes and the beer concentrations of ethyl hexanoate, ethyl octanoate and ethyl decanoate.

**Gene**	**Ethyl hexanoate**						**Ethyl octanoate**						**Ethyl decanoate**					
	**β coefficient**	**Standard Error of β**	***p*-value of β**	**R^2**	**Adjusted R^2**	***F*-test *p*-value**	**β coefficient**	**Standard Error of β**	***p*-value of β**	**R^2**	**Adjusted R^2**	***F*-test *p*-value**	**β coefficient**	**Standard Error of β**	***p*-value of β**	**R^2**	**Adjusted R^2**	***F*-test *p*-value**
Sc-*EHT1*	$-1.3 \cdot 10^{-4}$	$6.0 \cdot 10^{-5}$	0.059	0.87	0.83	$8 \cdot 10^{-4}$	$-1.4 \cdot 10^{-5}$	$1.1 \cdot 10^{-4}$	0.907	0.14	-0.10	0.58	$-7.0 \cdot 10^{-5}$	$4.2 \cdot 10^{-5}$	0.140	0.51	0.36	0.08
Se-*EHT1*	**$-3.8 \cdot 10^{-5}$**	$5.7 \cdot 10^{-6}$	$3 \cdot 10^{-4}$				$-1.1 \cdot 10^{-5}$	$1.1 \cdot 10^{-5}$	0.350				$4.0 \cdot 10^{-6}$	$4.0 \cdot 10^{-6}$	0.351			
Sc-*EEB1*	**$1.6 \cdot 10^{-5}$**	$5.6 \cdot 10^{-6}$	0.029	0.78	0.72	0.005	$6.1 \cdot 10^{-6}$	$7.8 \cdot 10^{-6}$	0.458	0.30	0.11	0.28	$3.7 \cdot 10^{-7}$	$3.8 \cdot 10^{-6}$	0.925	0.26	0.04	0.35
Se-*EEB1*	$-4.8 \cdot 10^{-6}$	$6.2 \cdot 10^{-6}$	0.465				$-4.0 \cdot 10^{-6}$	$8.6 \cdot 10^{-6}$	0.658				$4.6 \cdot 10^{-6}$	$4.2 \cdot 10^{-6}$	0.304			

A **bolded** β coefficient suggests a significant ($p < 0.05$) correlation between the transcription of that gene and that aroma compound.

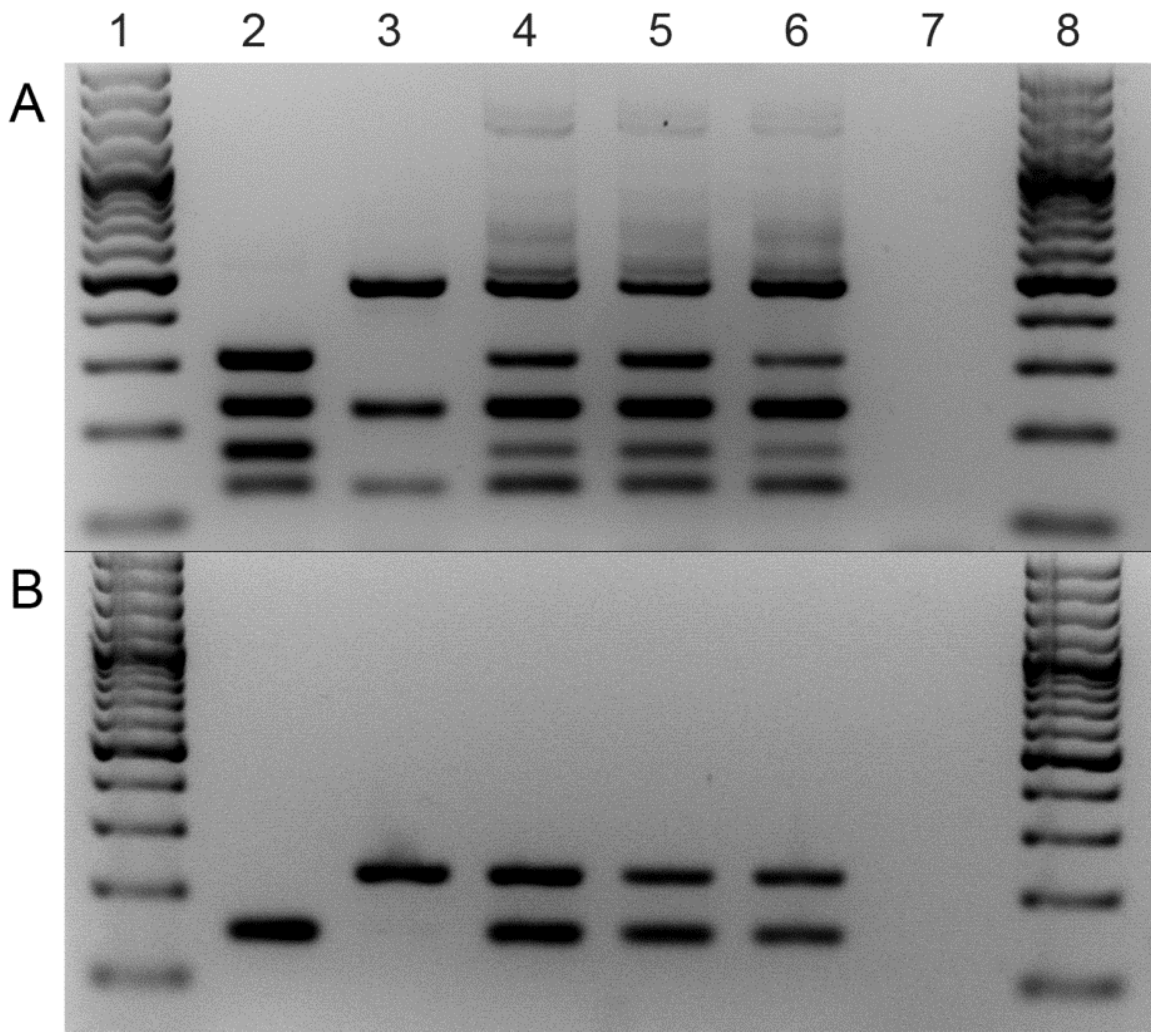

Fig. S1. Confirmation of hybridization by (**A**) rDNA ITS PCR and RFLP, and (**B**) amplification of *FSY1* and *MEX67* genes using species-specific primers. Lanes 1 & 8 100 bp DNA ladder, lane 2 *S. cerevisiae* A81062 parental strain, lane 3 *S. eubayanus* C12902 parental strain, lane 4 Hybrid C4, lane 5 Hybrid B3, lane 6 Hybrid A2, and lane 7 blank control.

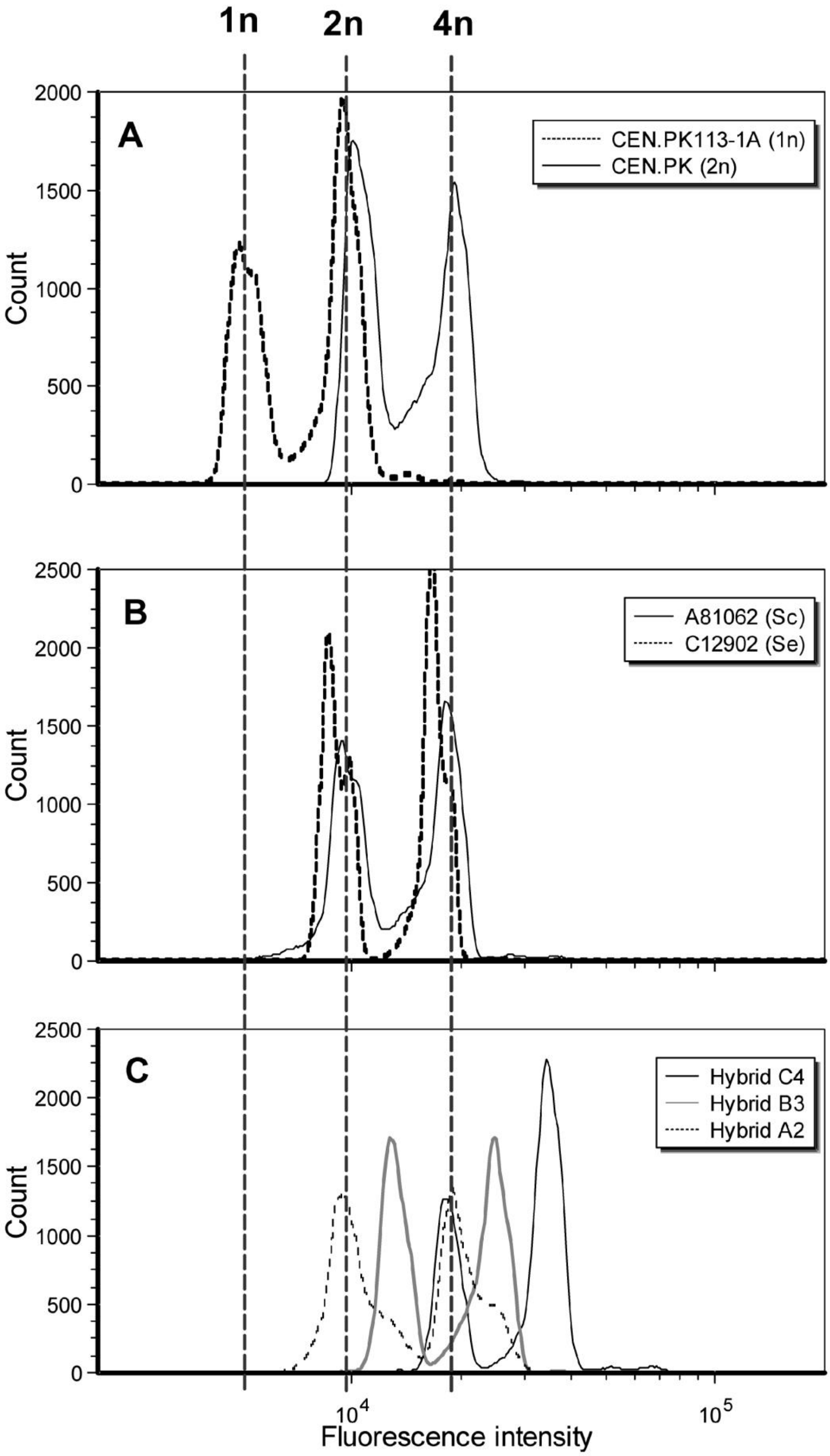

Fig. S2. DNA content of the (**A**) *S. cerevisiae* haploid (CEN.PK113-1A) and diploid (CEN.PK) reference strains, (**B**) *S. cerevisiae* A81062 and *S. eubayanus* C12902 parent strains, and (**C**) Hybrid A2, B3 and C4 strains by flow cytometry. The dashed vertical lines represent the approximate fluorescence intensity corresponding to 1n, 2n and 4n DNA content.

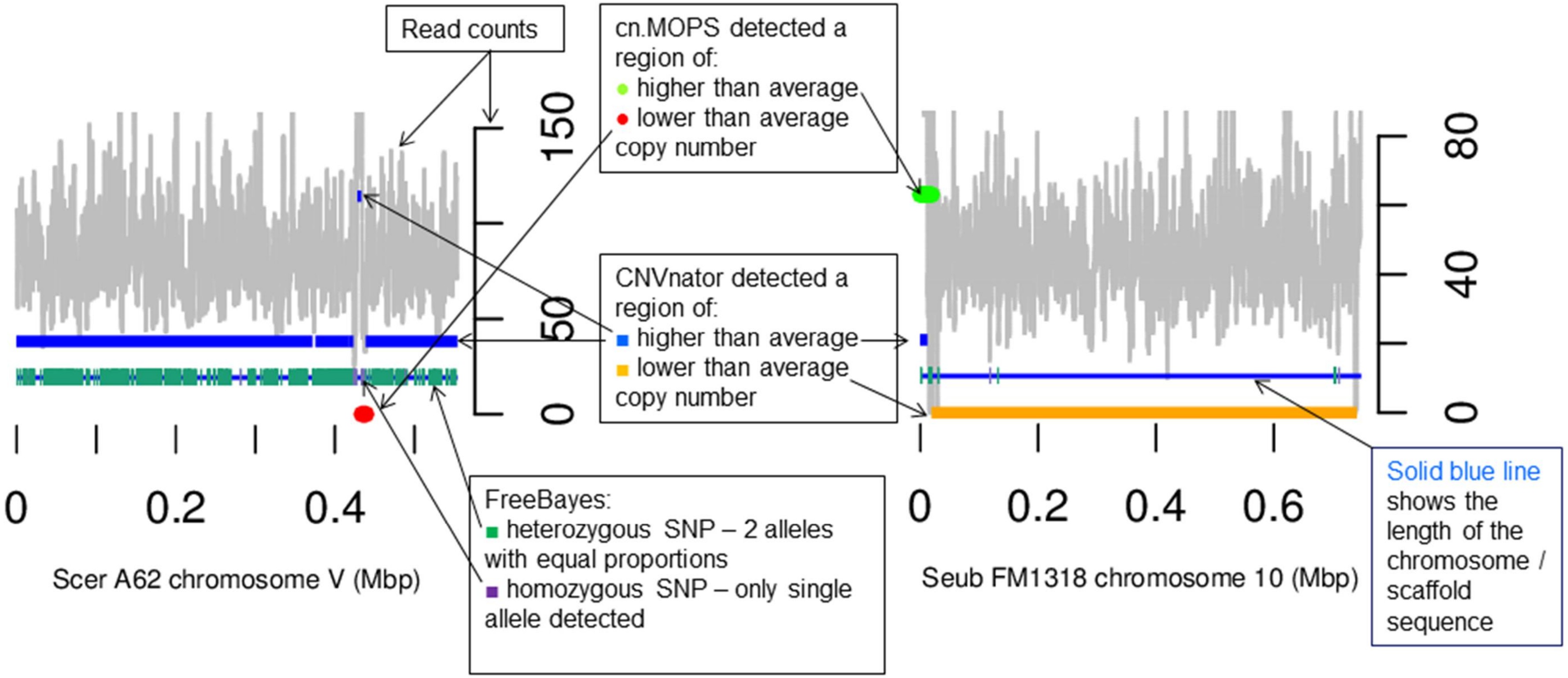

Fig. S3. Legend for the chromosome plots in Figures S4-S10. The x-axis shows the chromosomal position (Mbp), while the y-axis shows read count. The horizontal thin solid blue line shows the length of the chromosome/scaffold sequence. The SNPs that were detected by FreeBayes are displayed on top of this solid blue line in either green (heterozygous SNP) or purple (homozygous SNP). The regions that CNVnator detected as having higher or lower than average copy numbers are visualized with a thick blue or orange lines, respectively. The regions that cn.MOPS detected as having higher or lower than average copy numbers are visualized with green or red dots, respectively.

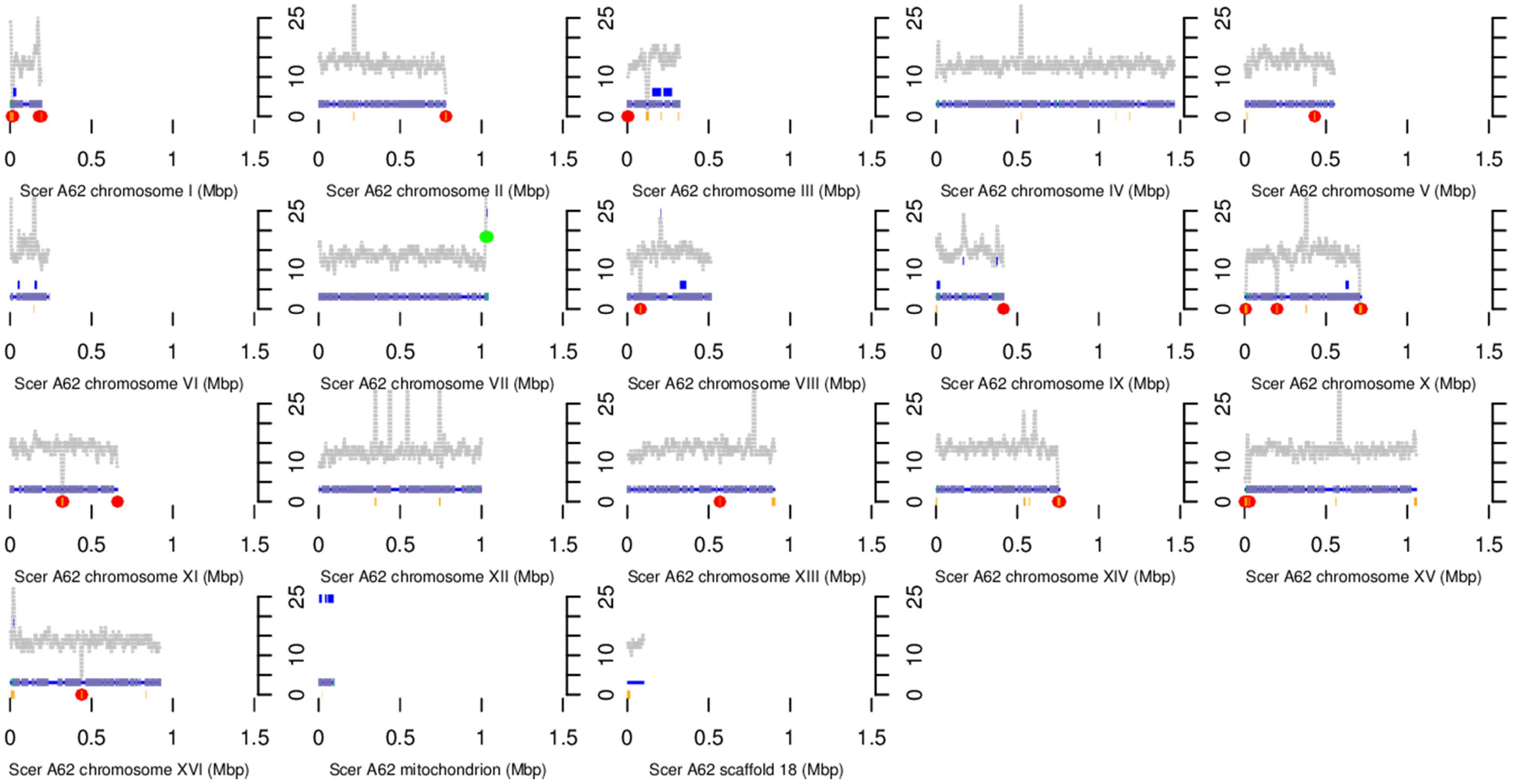

Fig. S4. Chromosome plots of the *S. cerevisiae* subgenome in the allodiploid Hybrid A2 strain. For more a more detailed legend, see Figure S3.

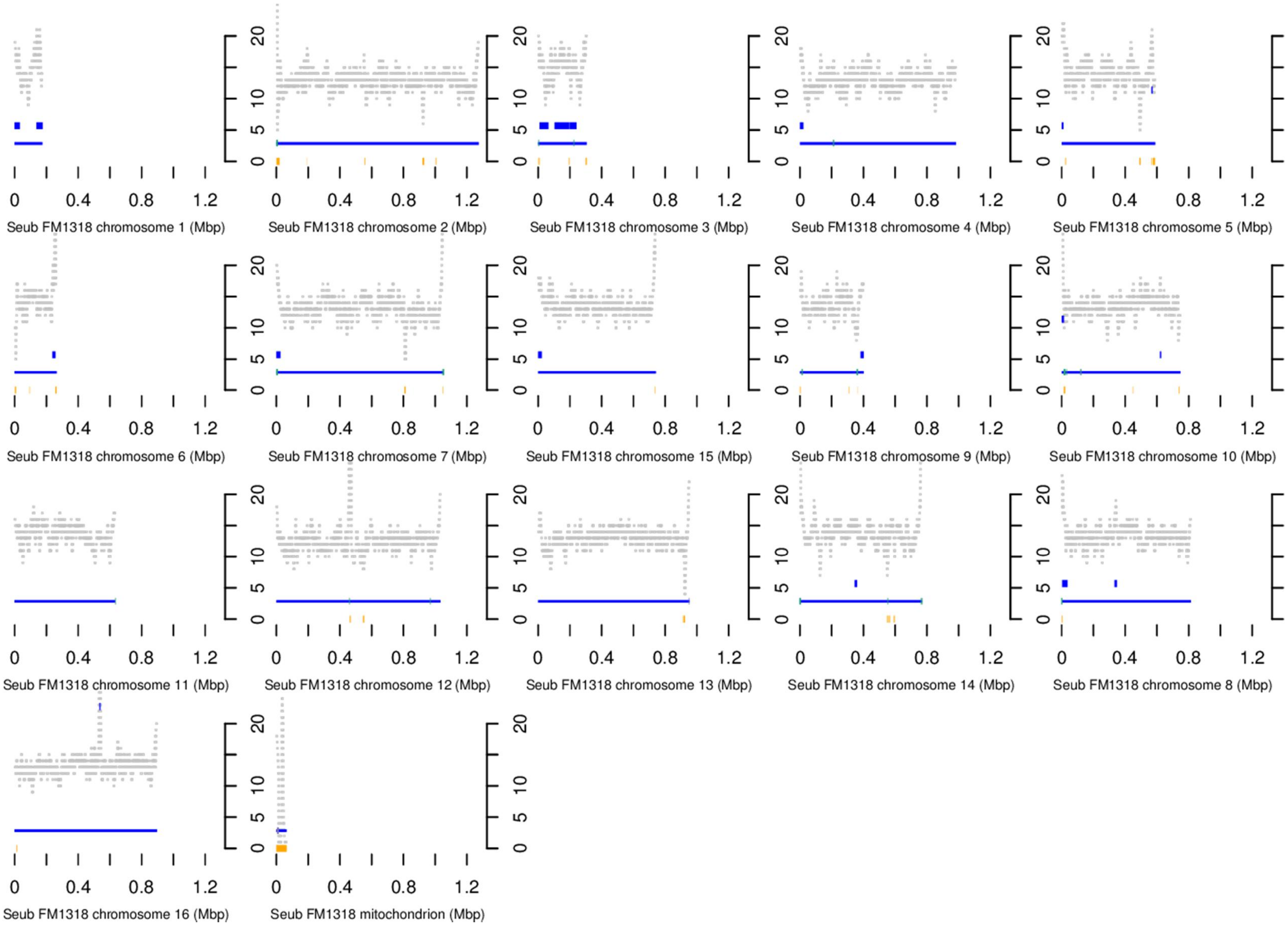

Fig. S5. Chromosome plots of the *S. eubayanus* subgenome in the allodiploid Hybrid A2 strain. For more a more detailed legend, see Figure S3.

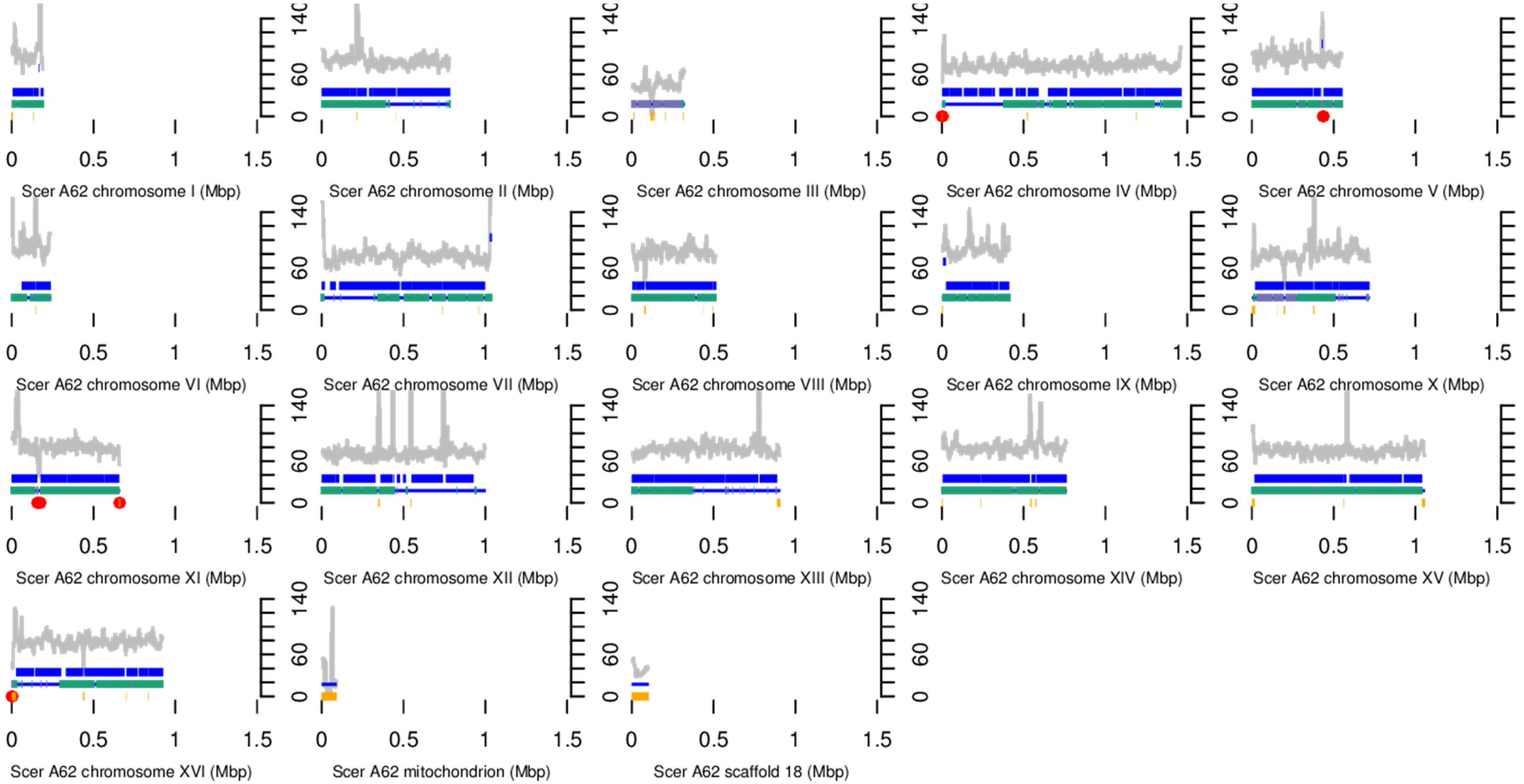

Fig. S6. Chromosome plots of the *S. cerevisiae* subgenome in the allotriploid Hybrid B3 strain. For more a more detailed legend, see Figure S3.

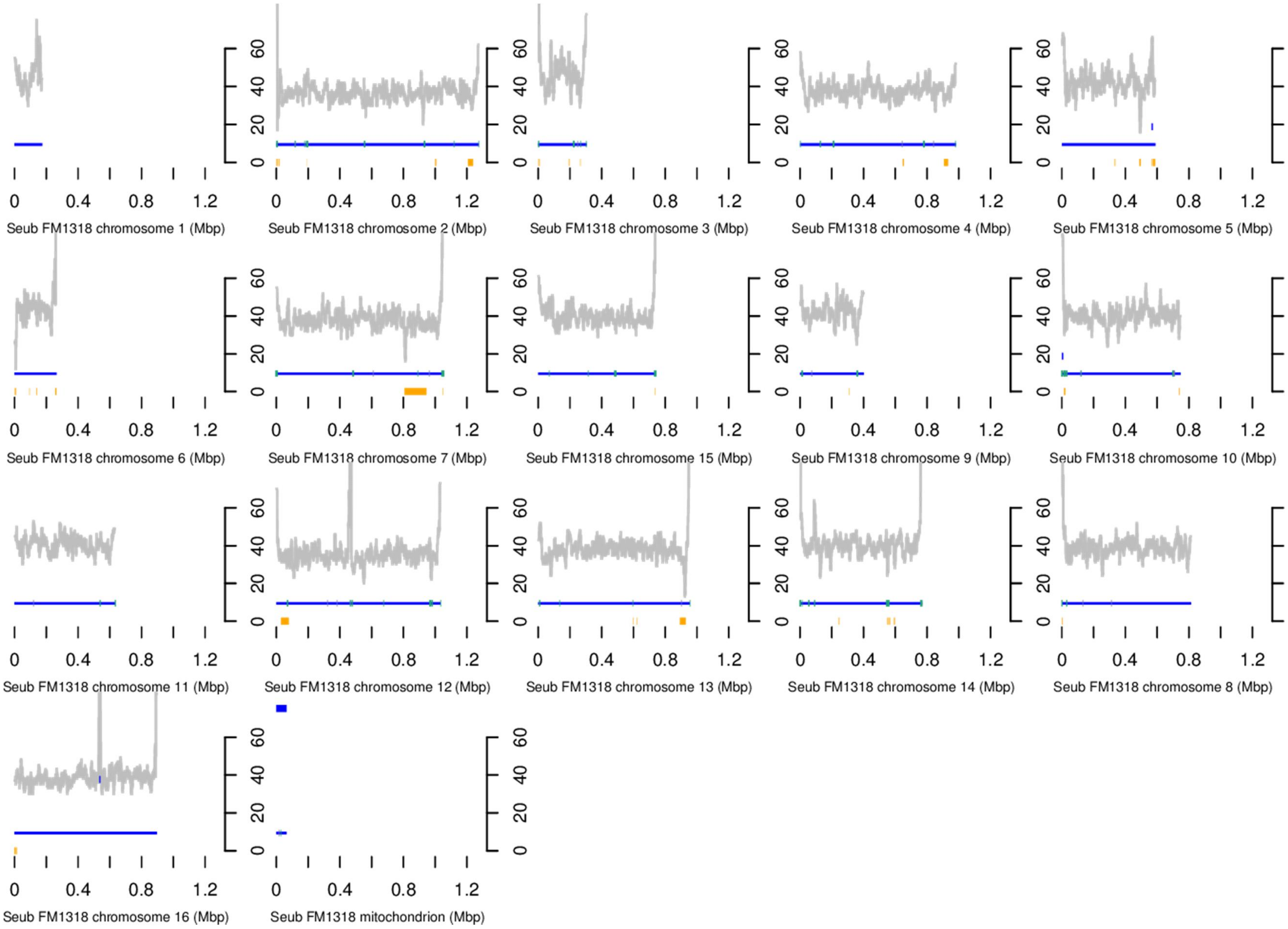

Fig. S7. Chromosome plots of the *S. eubayanus* subgenome in the allotriploid Hybrid B3 strain. For more a more detailed legend, see Figure S3.

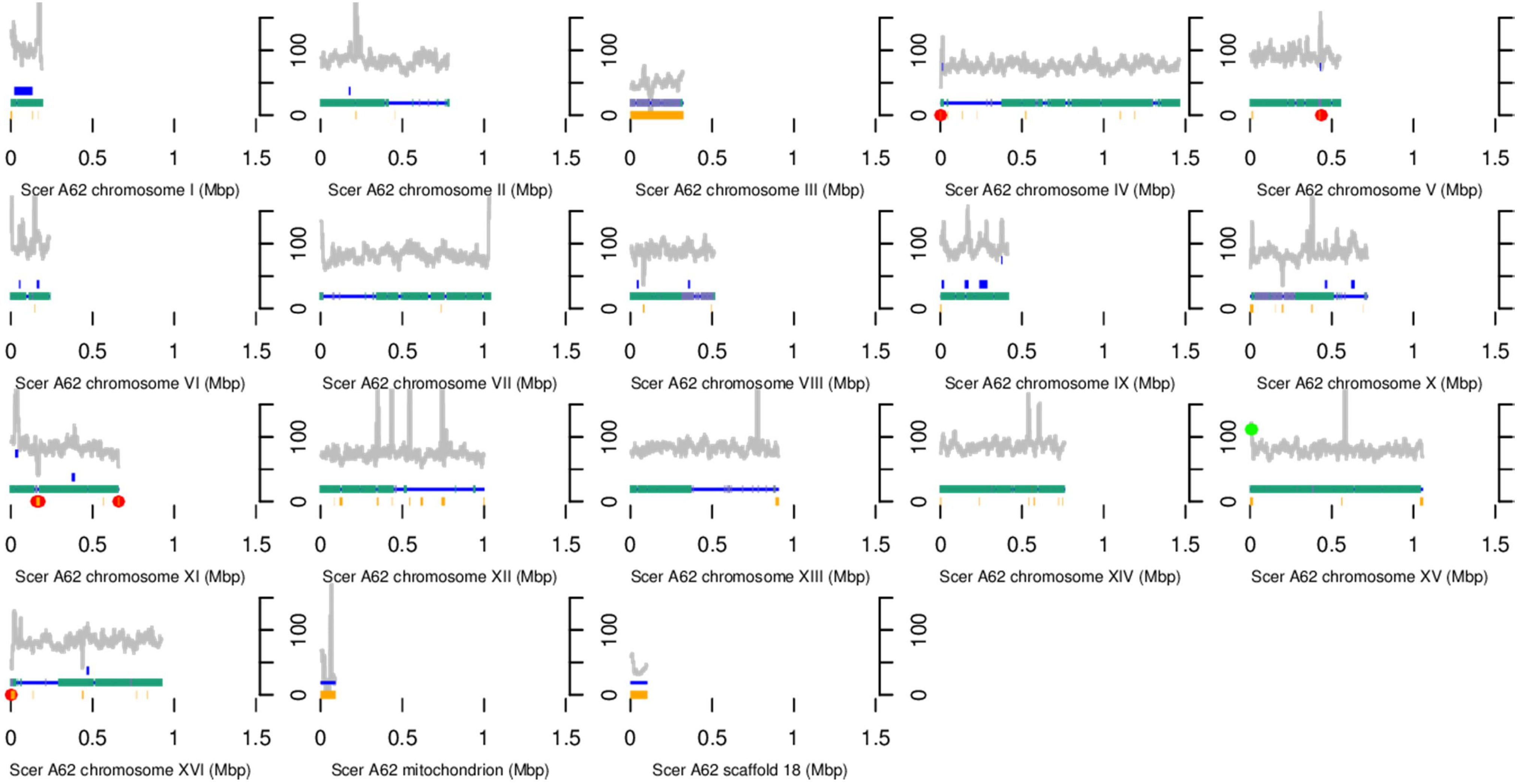

Fig. S8. Chromosome plots of the *S. cerevisiae* subgenome in the allotetraploid Hybrid C4 strain. For more a more detailed legend, see Figure S3.

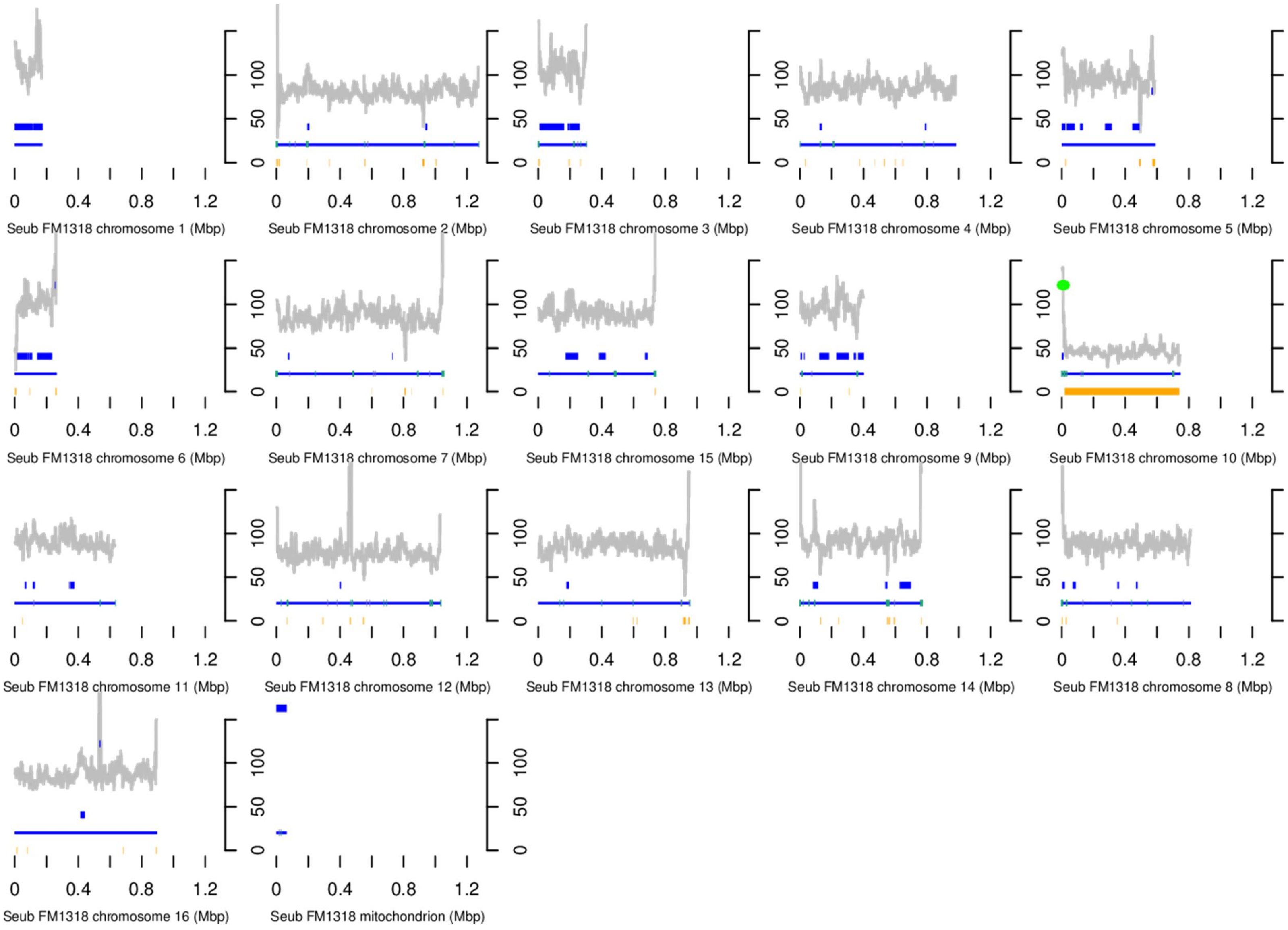

Fig. S9. Chromosome plots of the *S. eubayanus* subgenome in the allotetraploid Hybrid C4 strain. For more a more detailed legend, see Figure S3.

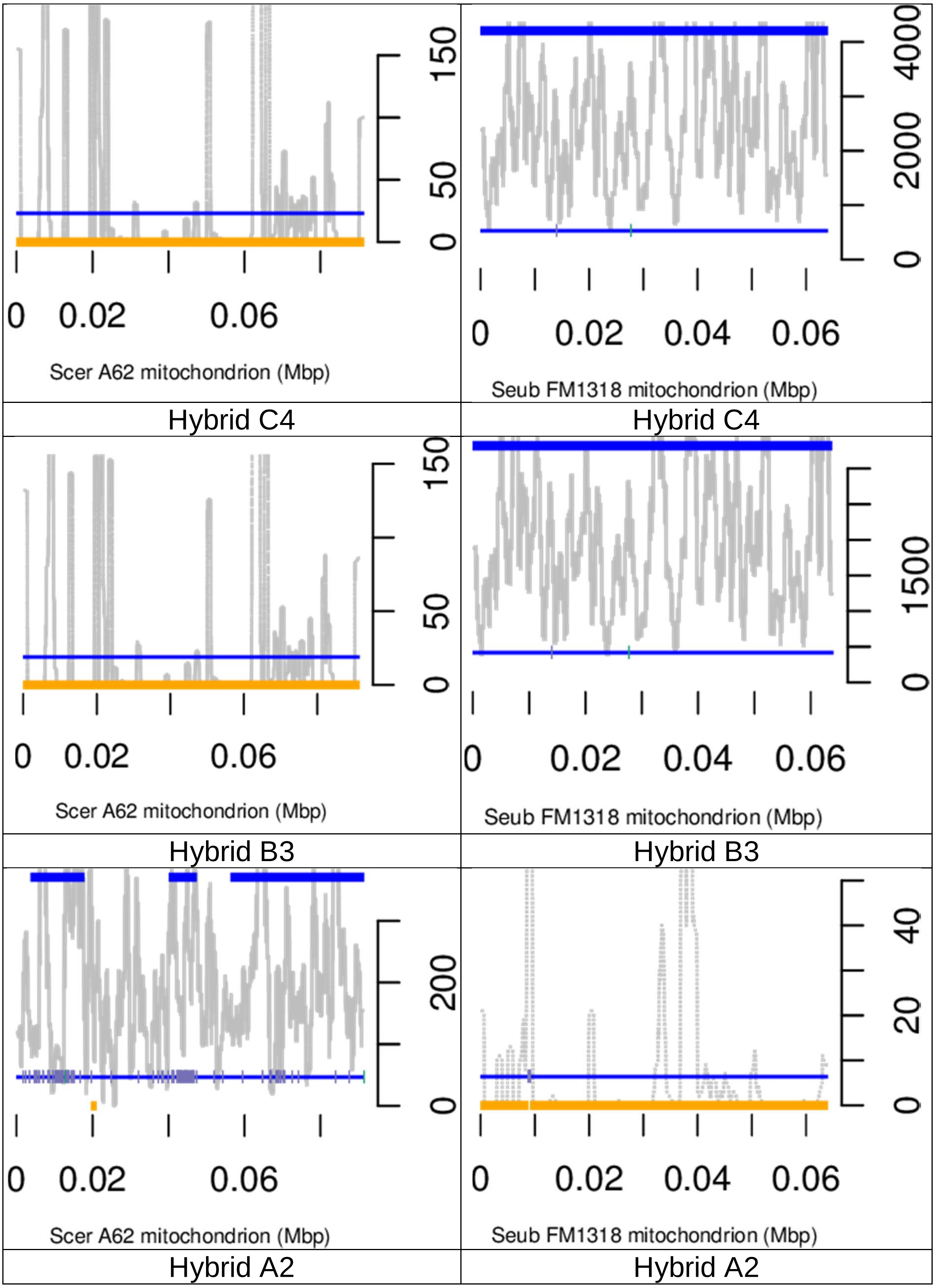

Fig. S10. Mitochondrial genomes of the three hybrid strains. For more a more detailed legend, see Figure S3.

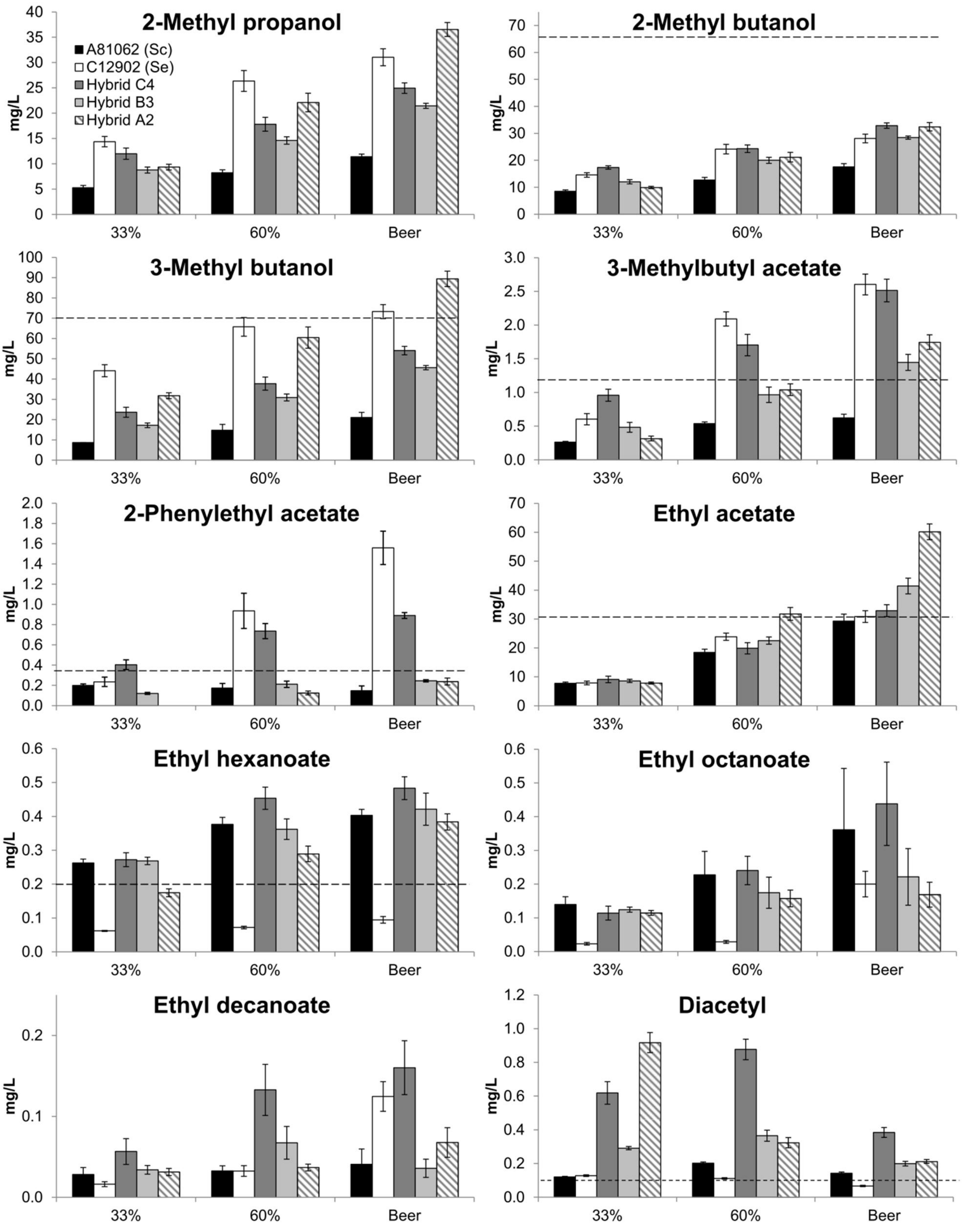

Fig. S11. The concentrations of aroma compounds in the wort (33% and 60% attenuation) and beers fermented from the 15 °P wort with the hybrid and parent strains (mg L^{-1}). Where visible, the dashed line represents the typical flavour threshold (Meilgaard 1982). Values are means from two independent fermentations and error bars where visible represent the standard deviation.

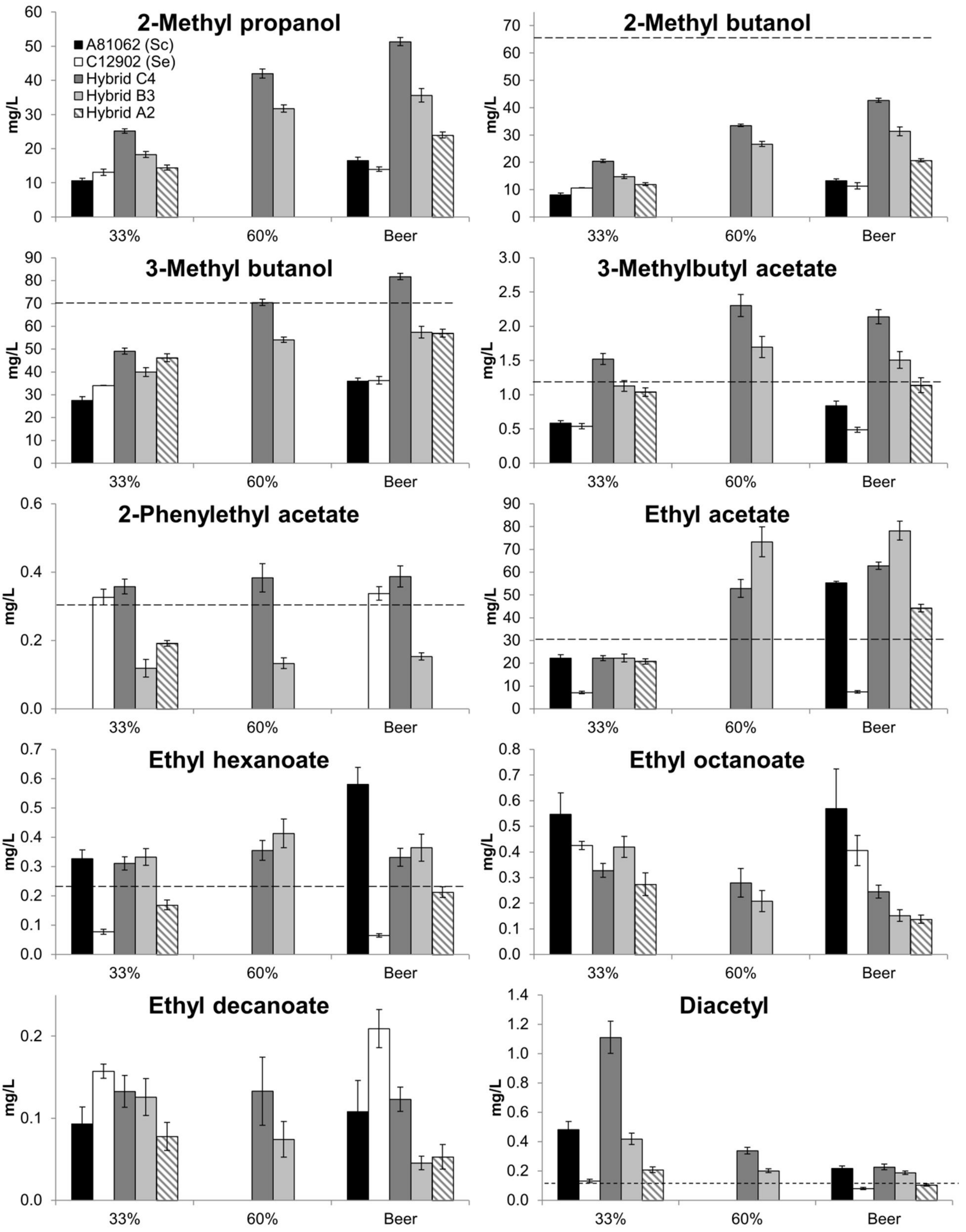

Fig. S12. The concentrations of aroma compounds in the wort (33% and 60% attenuation) and beers fermented from the 25 °P wort with the hybrid and parent strains (mg L^{-1}). Samples at 60% attenuation were only taken from fermentations with Hybrid C4 and Hybrid B3. Where visible, the dashed line represents the typical flavour threshold (Meilgaard 1982). Values are means from two independent fermentations and error bars where visible represent the standard deviation.

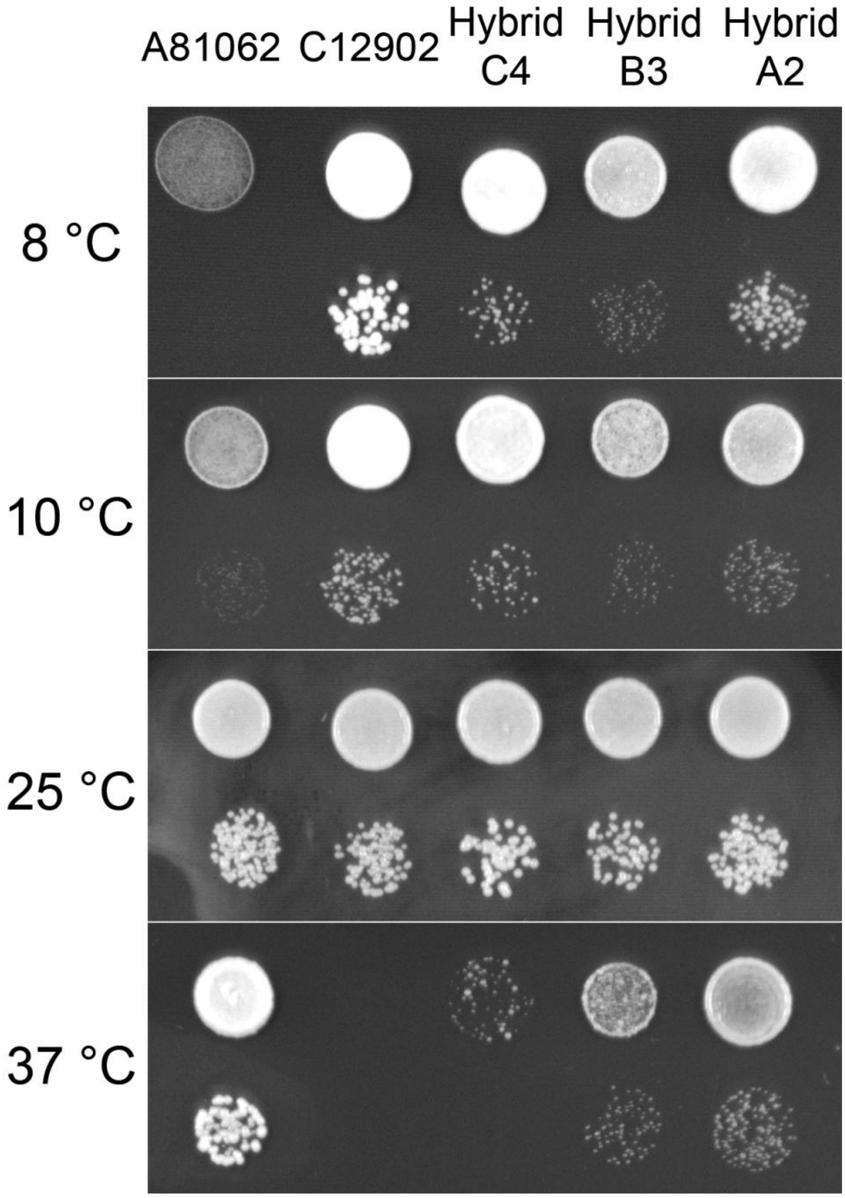

Fig. S13. Yeast growth assay showing a broader temperature range of growth for the hybrid strains compared to the parent strains. 10-fold serial dilutions of the yeast strains were spotted onto YPM agar plates and incubated at four different temperatures: 8 °C (12 days), 10 °C (8 days), 25 °C (2 days) and 37 °C (4 days).
